# Green Brönsted acid ionic liquids as novel corrosion inhibitors for carbon steel in acidic medium

**DOI:** 10.1038/s41598-017-07925-y

**Published:** 2017-08-18

**Authors:** Shuyun Cao, Dan Liu, Peng Zhang, Lixia Yang, Peng Yang, Hui Lu, Jianzhou Gui

**Affiliations:** 1 State Key Laboratory of Separation Membranes and Membrane Processes, School of Material Science and Engineering, Tianjin Polytechnic University, Tianjin, 300387 P.R. China; 2 School of Environmental and Chemical Engineering, Tianjin Polytechnic University, Tianjin, 300387 P.R. China; 3 Key laboratory of Structure-Based Drug Design and Discovery of Ministry Education, Shenyang Pharmaceutical University, Shenyang, 110016 P.R. China; 4 State Key Laboratory of Catalysis, Dalian Institute of Chemical Physics, Chinese Academy of Sciences, Dalian, 116023 P.R. China

## Abstract

New ionic liquids with multiple Brönsted acid sites were synthesized in ≥98% yield, and their inhibiting properties for the corrosion of carbon steel in 0.5 M HCl solution had been evaluated using electrochemical impedance spectroscopy, potentiodynamic polarization and weight loss method, finally the possible inhibiting mechanism was proposed according to UV–visible spectroscopic measurements and surface analysis including SEM and XPS techniques. The designed cation structure of Brönsted acid ionic liquids (BAILs), with one phenyl and two imidazolium rings, makes them good mixed-type inhibitors via the adsorption of BAILs on the steel surface to suppress both anodic and cathodic processes, obeying Langmuir adsorption isotherm. As potential acid catalysts, BAILs show nice corrosion inhibiting performance in acidic medium regardless of their Brönsted acidity, which is of great significance to enlarge the industry applications of BAILs.

## Introduction

Ionic liquids (ILs) as green molten salts have attracted great attention in the past decades, widely used in various applications such as catalysis, synthesis, lubrication and electrochemistry, etc.^1–3^. The various ILs with different physical and chemical properties can be designed by selecting the proper cation/anion combinations to meet different needs^4^. Due to the existence of strong Brönsted acid sites, Brönsted acid ionic liquids (BAILs) have their unique acidic characters, including high acid strength and controllable acid density, which have made them well documented^5–7^.

In our previous work, we have focused on the synthesis of task-specific ionic liquids, especially BAILs, and their applications as the solvents and catalysts in many acid-catalyzed reactions such as oxidation, alkylation, esterification and so on^8–11^, which demonstrates nice replacements of industrial-used conventional acid catalysts, such as HCl, H_2_SO_4_ and so on. The BAILs in this study contain multiple Brönsted acid sites, and are expected to be nice acid catalysts according to our previous work^12^.

Industrial utilization of acid catalysts usually inevitably involves interactions with containers, pipes, and equipment, and finally may lead to severe corrosion problems. It is well known that hydrochloric acid and sulfuric acid are strong corrosive acids in spite of good catalytic performance. How about the BAILs? The corrosion behaviour of some BAILs to various metal or alloys is quite different from conventional acid e.g., HCl, H_2_SO_4_^13^. We have studied the corrosion behaviour of mild steel in the BAIL 3-methyl-1-(4-sulfonic acid) butyl imidazolium bisulphate previously, and interestingly nice electrochemical passivation to mild steel could be obtained in spite of the acidity of the BAIL^14^. It is promising to find that BAILs show good inhibition efficiency to carbon steel in HCl aqueous solution, which will greatly increase the potential for industrial applications of BAILs.

Some ILs have been reported as corrosion inhibitors. Motsie E. Mashuga investigated ionic liquid namely 1-hexyl-3 methylimidazolium hexafluorophosphate [HMIM][PF_6_] as an inhibitor for the corrosion of mild steel in 1 M HCl solution, with the inhibition efficiency above 70%^15^; Xin Zhou reported 1-hexyl-3-methy limidazolium tetrafluoroborate [BMIM]BF_4_ as an inhibitor for the corrosion of carbon steel in alkaline chloride solution with the highest inhibition efficiency up to above 85%^16^. However, the anions BF_4_^−^ and PF_6_^−^ contain halogen atoms, which may cause serious concerns at certain conditions^17^. Due to the designable character, environmental-friendly BAILs without halide atoms are highly pursued for good inhibition efficiency.

Generally, most inhibitors are organic compounds containing a π system and/or heteroatoms e.g., sulfur, nitrogen, oxygen and phosphorus atoms, which can be adsorbed on the metal surface and reduce the corrosion rate^16, 18, 19^. In this study, the introduction of carboxyl acid in the side chains of imidazolium rings and anionic HSO_4_^−^ & H_2_PO_4_^−^ endows BAILs good acid catalytic function, which will be included in our future work on the relationship of the structures and catalytic properties of BAILs; Meanwhile, BAILs with two imidazolium rings and one phenyl ring are expected to have good inhibiting capability^20^.

Due to the strong corrosivity of conventional acid including HCl and H_2_SO_4_, it is challenging to design new BAILs acting not only as good acid catalysts, but nice corrosion inhibitors in conventional acids as well. This is of great significance before their industry applications.

The inhibition capacity of BAILs for the corrosion of carbon steel in acidic medium is investigated using various methods, such as electrochemical impedance spectroscopy (EIS), potentiodynamic polarization (PDP) and weight loss (WL) measurements. Then the surface analysis of carbon steel before and after electrochemical tests have been conducted via scanning electron microscopy (SEM) and X-ray photoelectron spectroscopy (XPS). The UV-visible spectroscopic measurements are carried out to gain an insight into the possibility of forming the complex between Fe^2+^ ion released during the corrosion reaction and the BAIL in solution. Some thermodynamic parameters have been calculated, and the possible inhibition mechanism of BAILs has been predicted as well.

## Experimental Details

### Materials and sample preparation

**Figure 1:**
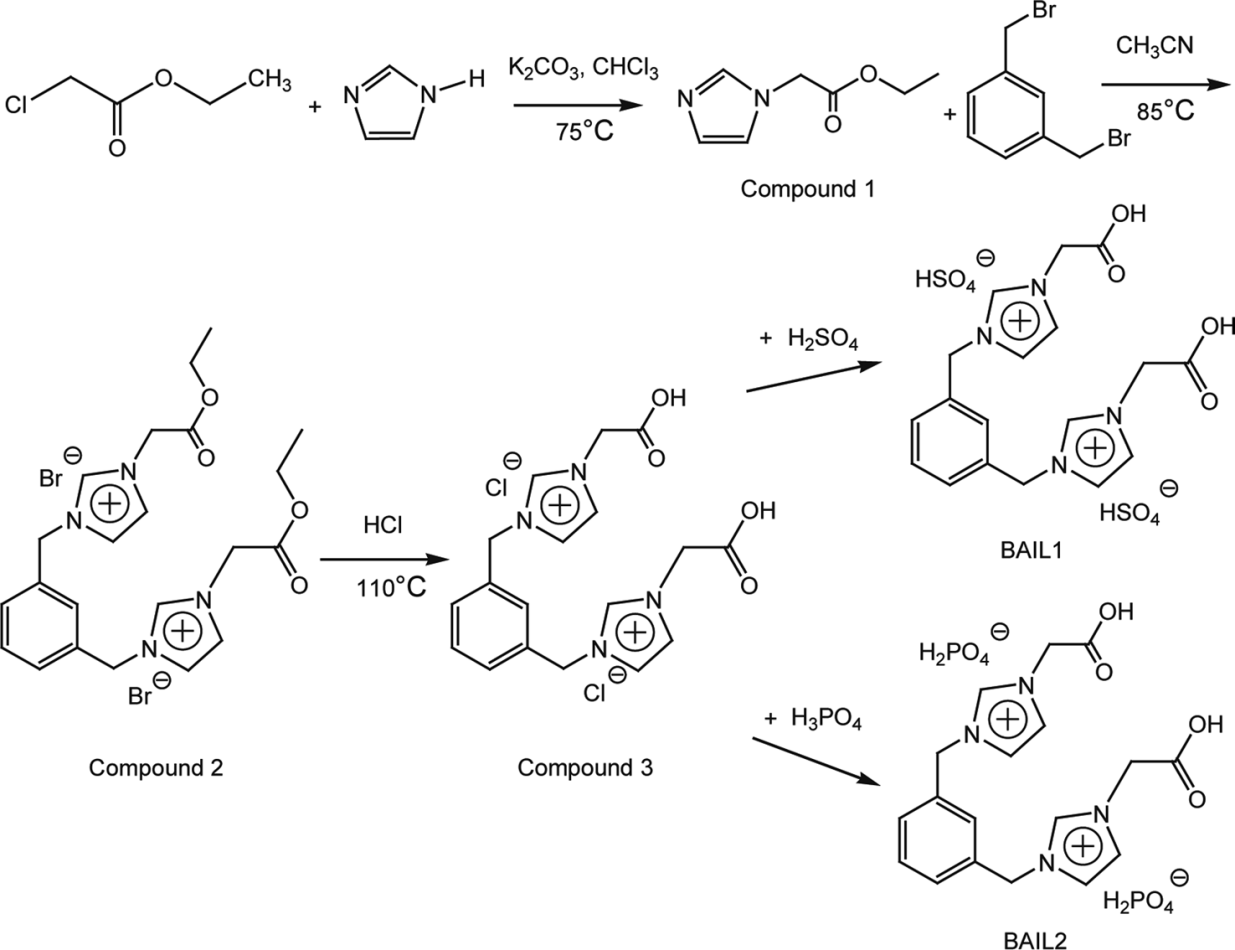
Synthesis schemes of BAIL1 and BAIL2.

All the chemical reagents involved in the experiments were analytical grade, and used without further purification unless otherwise stated. The synthesis schemes of corrosion-inhibiting-functionalized BAILs were shown in Fig. 1. Ethyl 1H-imidazole-1-acetate (Compound 1) was obtained by reacting combining imidazole and potassium carbonate in the presence of ethyl chloroacetate; and then ethyl imidazolium acetate reacted with 1, 3-bis(bromomethyl) benzene in acetonitrile to yield 1, 1′-(1, 4-phenylene bis(methylene)) bis(3-(2-ethoxy-2-oxoethyl)-1H-imidazol-3-ium) bromide (Compound 2); and the reaction of Compound 2 with 1 M aqueous HCl solution was carried out for 1, 1′-(1, 4-phenylenebis(methylene))bis(3-(carboxymethyl)-1H-imidazol-3-ium) chloride (Compound 3). Finally, the synthesis of targeting BAIL1 and BAIL2 (i.e., 1,1′-(1, 4-phenylenebis(methylene))bis(3-(carboxymethyl)-1H-imidazol-3-ium) bisulfate or dihydrogen phosphate) was obtained by reacting one mole of Compound 3 with two moles of concentrated sulfuric or phosphoric acid in dichloromethane, respectively. The detailed synthesis method was provided in the Supplementary Information. All of the above BAILs were characterized by ^1^H NMR, ^13^C NMR, and Fourier transform infrared spectroscopy (FTIR), and the results were included in the Supplementary Information (Figs S1–S6). The ^1^H and ^13^C NMR were measured on a Bruker AVANCE III 600 MHz spectrometer. The FTIR spectra were recorded with a Perkin-Elmer FTIR model 1600.

The aggressive medium of 0.5 M HCl solution was prepared by diluting the stock solution (37% HCl) using bi-distilled water. The inhibited solution with various concentrations (0, 0.25, 0.5, 0.75 and 1 mM, respectively) of the BAILs were prepared by adding suitable amount of as synthesized BAILs into 250 mL volumetric flask and bring to volume by 0.5 M HCl solution.

The composition (wt%) of the carbon steel coupons is C: 0.42~0.50%, Si: 0.17~0.37%, Mn: 0.50~0.80%, Cr: ≤0.25%, Ni: ≤0.30%, Cu: ≤0.25%, Fe for balance. The exposed surface area of carbon steel used in the electrochemical experiments was 1 cm^2^, while the rest was embedded by epoxy resin. Besides, the dimension of steel specimens for weight loss experiments was 4.0 × 1.2 × 0.2 cm. Prior to each measurement, the surface of carbon steel specimens was successively abraded with abrasive paper from 360 up to 2000 grit size, then rinsed with bi-distilled water, degreased with anhydrous ethanol, and finally dried at room temperature.

### Electrochemical tests

All electrochemical measurements were performed with PARSTAT 2273 electrochemical workstation (Princeton Applied Research) in a conventional three-electrode cell with its electrolyte volume of 200 mL. The carbon steel was used as the working electrode (the exposed surface area is 1 cm^2^), a saturated calomel electrode (SCE) as the reference electrode, and a graphite electrode as the counter electrode. Before each EIS and subsequent PDP experiment, the steel surface was immersed in the 0.5 M HCl solution without or with different concentrations of BAIL1 or BAIL2 for 30 min until a steady state of the open circuit potential (OCP). All measurements were carried out in non-deaerated solutions and performed using aqueous thermostat bath.

The EIS experiments were performed with a 10 mV amplitude signal in the frequency range of 100 kHz–50 mHz. The inhibition efficiency (*IE*_EIS_%) was analyzed by EIS experiments through the follow equation^15^:

1 $$I{E}_{{\rm{E}}{\rm{I}}{\rm{S}}}{\rm{ \% }}=({R}_{{\rm{c}}{\rm{t}}}-{R}_{{\rm{c}}{\rm{t}}({\rm{i}}{\rm{n}}{\rm{h}})})/{R}_{{\rm{c}}{\rm{t}}}\times 100$$

where *R*_ct_ and *R*_ct(inh)_ are the values of charge transfer resistance without or with inhibitors for carbon steel in 0.5 M HCl solution, respectively.

The PDP measurements were carried out in the potential ranging from −150 mV to 250 mV versus the OCP with a scan rate of 0.5 mV s^−1^. The inhibition efficiency (*IE*_PDP_%) was calculated by extrapolation of cathodic (*β*_c_) and anodic (*β*_a_) branches of Tafel curves using the equation^15^:

2 $$I{E}_{{\rm{PDP}}} \% =({i}_{{\rm{corr}}}\,\mbox{--}\,{i}_{{\rm{inh}}})/{i}_{{\rm{corr}}}\times 100$$

where *i*_corr_ and *i*_inh_ are the corrosion current densities of carbon steel in 0.5 M HCl solution without or with inhibitors, respectively.

### Weight loss measurements

Weight loss is a more reliable technique for the determination of corrosion rates and inhibition efficiency than electrochemical techniques^21^. The weight loss of carbon steel coupons was measured over 24 h of immersion in 100 mL HCl solutions without or with different concentrations of BAIL1 and BAIL2 at room temperature, respectively. The corrosion rate (*CR*), the inhibition efficiency (*IE*_WL_%) and the degree of surface coverage (*θ*) were calculated by the following the equations:

3 $$CR({\rm{m}}{\rm{g}}\cdot {{\rm{c}}{\rm{m}}}^{-2}\cdot {{\rm{h}}}^{-1})={\rm{\Delta }}W/(A\times t)$$

4 $$I{E}_{{\rm{WL}}} \% =(C{R}_{0}-C{R}_{{\rm{inh}}})/C{R}_{0}\times 100$$

5 $$\theta =I{E}_{{\rm{WL}}}/100$$

where Δ*W* is the weight loss in mg, *A* is the area of the carbon steel exposed in solution in cm^2^, *t* is exposure time in hours, and *CR*_0_ and *CR*_inh_ are the corrosion rate in uninhibited and inhibited 0.5 M HCl solution, respectively.

### UV–visible spectrophotometric studies

For optical characterization, UV–visible absorption spectrophotometric measurements were carried out within the working range of 200–600 nm. The absorption spectra of all the tested solutions were recorded using UV–vis Spectrophotometer (Evolution 300, Thermo), and determined with uninhibited 0.5 M HCl solution as reference.

### Surface analysis

After some electrochemical tests including OCP- EIS- PDP tests, the steel electrodes were cleaned with bi-distilled water and acetone, and dried by blowing with cool air for further surface analysis. The surface morphologies and chemical compositions of fresh or corroded samples were examined using a scanning electron microscope (SEM, Phenom prox) and XPS (ESCALAB 250, VG Company), respectively. XPS had been performed with a monochromatic Al *K*α (1486.6 eV) radiation source, and the binding energies were referenced to the C 1s peak (284.6 eV) of carbon. The collected XPS data were dealt with XPS PEAK Version 4.1 software for Shirley background subtraction, fitting and deconvolution of the peaks.

## Results and Discussion

### Open circuit potential

**Figure 2:**
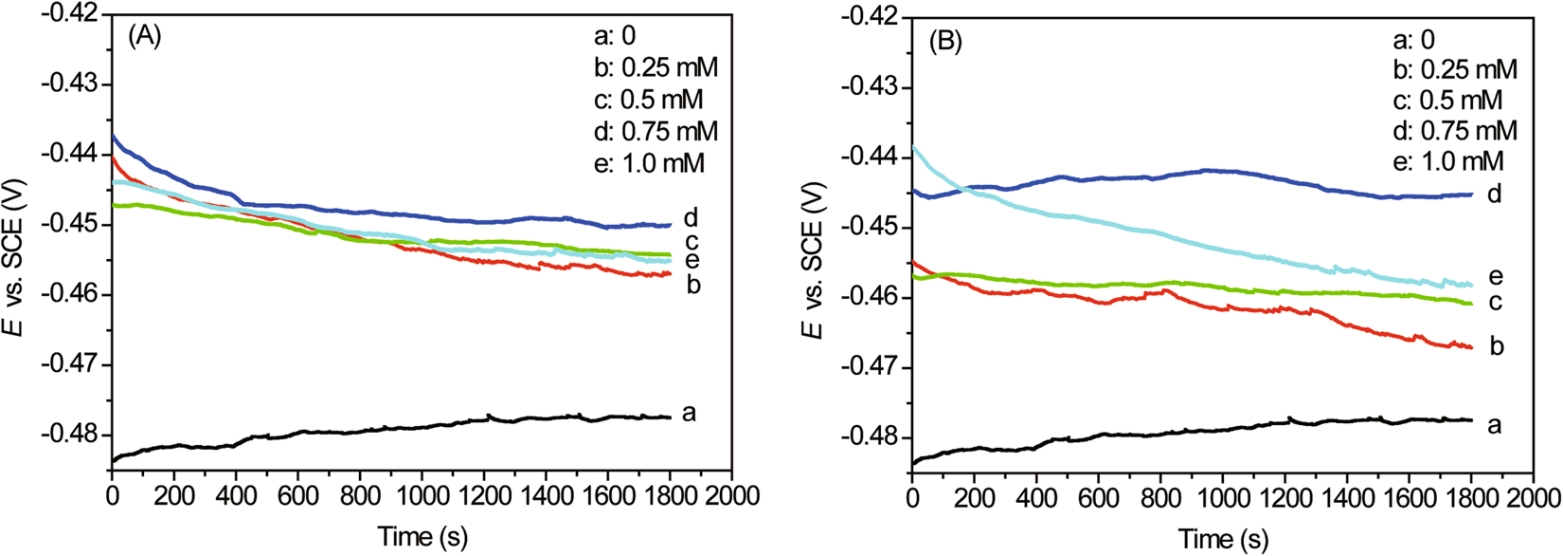
Time variation of the open circuit potential (OCP) for carbon steel in 0.5 M HCl solution without/with different concentrations of BAIL1 (**A**) and BAIL2 (**B**).

The results of open circuit potential studies were shown in Fig. 2. As compared with the blank, it is obvious that the addition of BAILs shifts the stable potential (*E*_OCP_) towards less negative value, with a distinct shift (about 27–32 mV) at the inhibitor BAIL concentration of 0.75 mM, which may be attributed to the formation of a protective film on the electrode surface^22^.

### Electrochemical impedance spectroscopy

**Figure 3:**
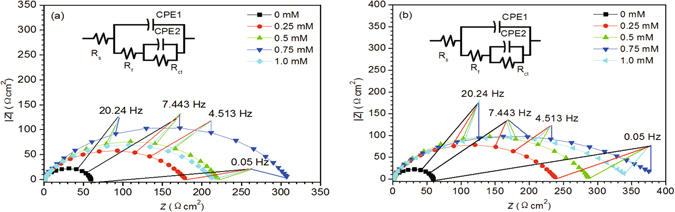
Nyquist plots for carbon steel in 0.5 M HCl solution without/with different concentrations of BAIL1 (**a**) and BAIL2 (**b**).

**Figure 4:**
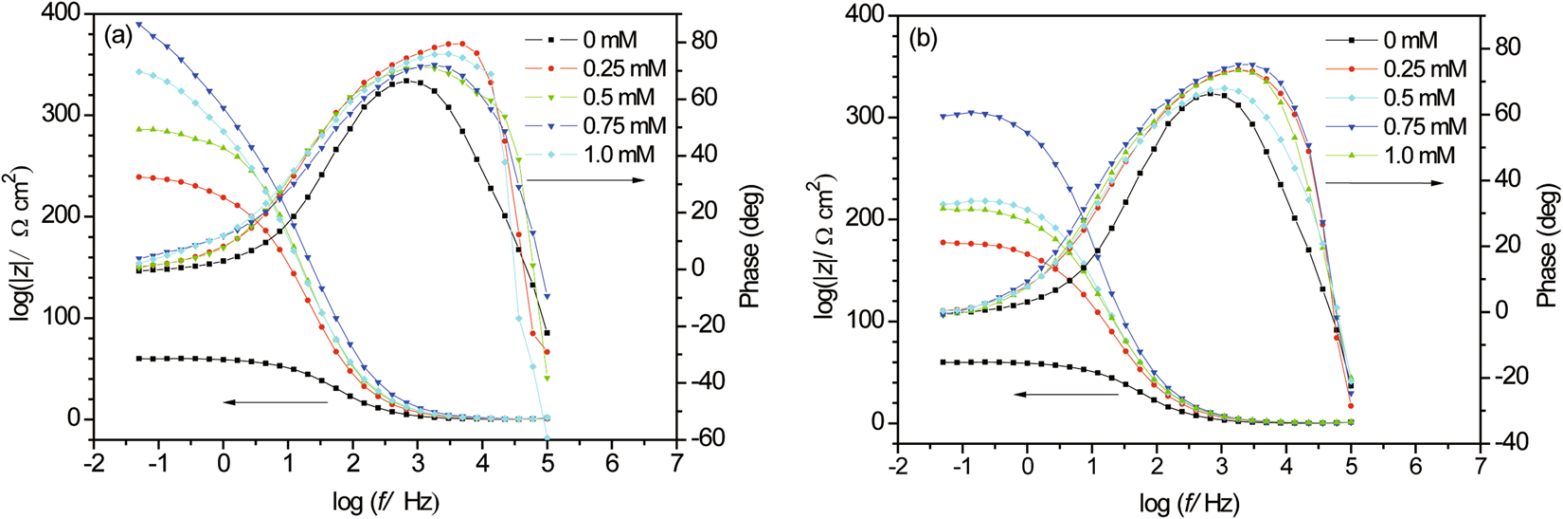
Bode plots for carbon steel in 0.5 M HCl solution without/with different concentrations of BAIL1 (**a**) and BAIL2 (**b**).

The representative Nyquist and Bode plots of carbon steel immersed in 0.5 M HCl solution without or with different concentrations of BAILs at the OCP are shown in Figs 3 and 4, respectively.

As can be seen from Fig. 3, all the impedance spectra in EIS tests show depressed semicircles, and the deviation to a perfect semicircle may be attributed to inhomogeneity and roughness of the electrode surface^23^. An increased radius of the impedance plot in the presence of BAILs suggests the enhanced inhibition efficiency, so the inhibition efficiency (*IE*_EIS_%) increases with the concentration of BAILs within the range of 0.25 to 0.75 mM, reaching its maximum value 80.4% (BAIL1) and 89.7% (BAIL2), and then decreases when further increasing the concentration of BAILs.

The Bode plot in Fig. 4 shows the shifts of frequency at maximum phase angle to higher value in the presence of inhibitors, and two time constants could be observed in the presence of inhibitors. The equivalent circuit shown in the inset of Fig. 1 has been used for EIS data fitting. *R*_s_ is the solution resistance, the first time constant at high frequencies can be described to the film resistance (*R*_f_), which corresponds to the adsorption of inhibitor, and the other one at low frequencies is related to the charge transfer resistance (*R*_ct_), which can be ascribed to the electron transfer reactions occurring in the metal/solution interface. Generally, *CPE* representing a constant phase element is often used to replace the double layer capacitance (*C*_dl_) to give a more accurate fit^24^. *CPE*_1_ and *CPE*_2_ are constant phase elements correspond to the capacitance of the film formed on the electrode surface and the double layer capacitance at the interface between the steel and solution in the film pores. The impedance function of the *CPE* can be expressed as follows^25^.

6 $${Z}_{{\rm{CPE}}}=1/{Y}_{0}{(j\omega )}^{{\rm{n}}}$$

where *Y*_0_ is the *CPE* constant, *j* is the imaginary number (*j* ^2^ = −1), *ω* (*ω = *2π*f*) is the angular frequency, and *n* (−1 ≤*n* ≤1) is the phase shift related to the inhomogeneity on the steel surface. *n* is a phase shift. While *n* = 0, *CPE* denotes a resistance; for *n* = 1, a capacitance; for *n* = 0.5, a Warburg element; and for *n* = −1, an inductance^25^. So in this paper, the *CPE*_1_ is equal to the *C*_dl_. In addition, the double layer capacitance, *C*_dl_ is obtained by the following equation^26^:

7 $${C}_{{\rm{dl}}}={Y}_{0}{({\omega }_{{\rm{\max }}})}^{n-1}={Y}_{0}{(2\pi {f}_{{\rm{\max }}})}^{n-1}$$

where *f*_max_ is the frequency where the imaginary value reaches the maximum value on the Nyquist graph.

The fitted impedance parameters are listed in Table 1. The value of *R*_ct_ increases while the *C*_dl_ decreases with increasing the concentration of inhibitor within the range of 0.25 to 0.75 mM. The higher *R*_ct_ value (280.7 Ω cm^2^ for BAIL1; 379.7 Ω cm^2^ for BAIL2) has been found at optimum BAIL concentration of 0.75 mM, and the best inhibition efficiency (*IE*_EIS_%) for BAIL1 and BAIL2 are 88.0% and 91.1%, respectively. The increase of *R*_ct_ indicates the increase of inhibitor molecules adsorbed on the steel surface^27^, and the decrease in *C*_dl_ can be attributed to the decrease in local dielectric constant and/or the increase in the thickness of the double layer. Hence, these results may be attributable to the adsorption of BAIL forming protective adsorption layers on the steel surface^28, 29^. Further increasing the concentration of BAIL above 0.75 mM, the value of *R*_ct_ decreases while the *C*_dl_ increases with the concentration of BAIL, meanw hile the value of inhibition efficiency (*IE*_EIS_%) decreases accordingly, which can be attributed to the desorption of BAIL inhibitor due to extra concentration of BAIL. The addition of BAIL causes a decrease in the value of *n*2 at large, which may due to the surface inhomogeneity arising from the nonuniform film formed on the steel surface, and the random adsorption of BAIL molecules on the steel surface may be responsible for a decrease in steel surface homogeneity^30^.

**Table 1:** Electrochemical impedance parameters for carbon steel in 0.5 M HCl solution without/with different concentrations of BAIL1 and BAIL2.

Inhibitor	*C*_inh_ (mM)	*R*_s_ (Ω)	*C*PE		*R*_f_ (Ω cm^2^)	*CPE*		*R*_ct_ (Ω cm^2^)	*IE*_EIS_ (%)
			Y_0_ (μF cm^−2^)	*n1*		Y_0_ (μF cm^−2^)	*n2*		
BAIL1	0	0.81	44.77	1	25.01	368.9	0.7970	33.72	
	0.25	0.75	22.76	1	19.46	229.2	0.6548	159.7	78.9
	0.5	0.85	20.95	1	27.26	193.3	0.7143	192.9	82.5
	0.75	0.76	18.03	1	28.67	177.8	0.6921	280.7	88.0
	1.0	1.06	15.14	1	15.14	232.3	0.6643	197.8	83.0
	0	0.81	44.77	1	25.01	368.9	0.7970	33.72	
	0.25	0.83	20.89	1	31.29	237.3	0.6440	208.1	83.8
BAIL2	0.5	1.033	15.07	1	17.79	159.9	0.6708	270.3	87.5
	0.75	1.056	10.78	1	13.17	336.3	0.5075	379.7	91.1
	1.0	1.6290	16.40	1	35.44	343.7	0.5728	312.0	89.2

### Potentiodynamic polarization (PDP) measurements

**Figure 5:**
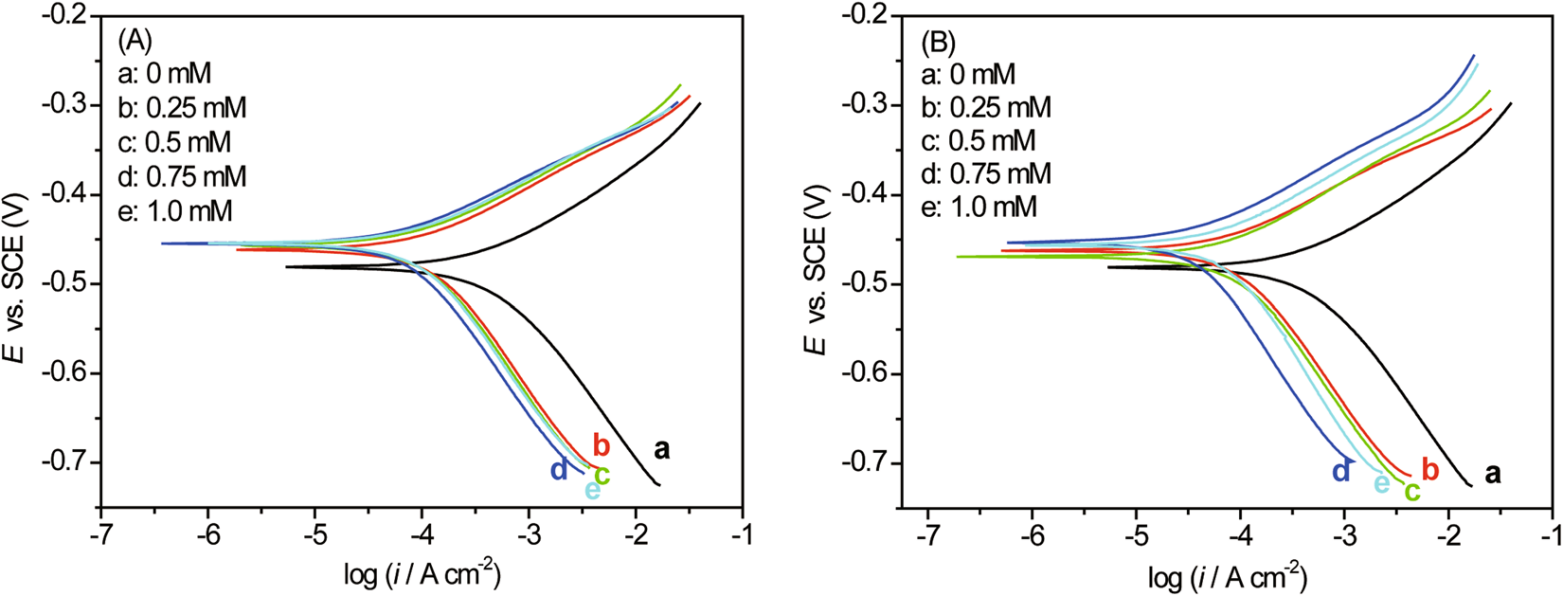
Potentiodynamic polarization (PDP) plots for carbon steel in 0.5 M HCl solution at a scan rate of 0.5 mV s^−1^ without/with different concentrations of BAIL1 (**A**) and BAIL2 (**B**).

The potentiodynamic polarization curves for carbon steel in 0.5 M HCl solution without or with different concentrations of BAILs are shown in Fig. 5, and the respective electrochemical parameters {i.e., corrosion current density (*i*_corr_), corrosion potential (*E*_corr_), anodic Tafel slope (*β*_a_), cathodic Tafel slope (*β*_c_), and inhibition efficiency (*IE*_PDP_%)} extracted from the corresponding Tafel plots (*E*_corr_ ± 100 mV) are given in Table 2.

**Table 2:** The potentiodynamic polarization parameters for carbon steel in 0.5 M HCl solution without/with different concentrations of BAIL1 and BAIL2.

Inhibitor	*C*_inh_ (mM)	*E*_OCP_ (mV/SCE)	*E*_corr_ (mV/SCE)	*i*_corr_ (μA cm^−2^)	*β*_c_ (mV dec^−1^)	*β*_a_ (mV dec^−1^)	*IE*_PDP_ (%)
BAIL1	0	−477	−481	366.4	149.6	86.2	
	0.25	−457	−462	115.8	159.4	72.8	68.4
	0.5	−454	−456	91.87	152.9	67.9	74.9
	0.75	−450	−455	67.47	153.2	64.9	81.6
	1.0	−454	−454	89.1	161.9	68.3	75.7
BAIL2	0	−477	−481	366.4	149.6	86.2	
	0.25	−469	−462	77.07	141.0	68.5	78.9
	0.5	−461	−469	73.82	144.3	75.9	79.8
	0.75	−445	−453	38.61	180.4	69.1	89.5
	1.0	−458	−456	71.83	177.2	75.5	80.4

It is apparent from Fig. 5 that regardless of the concentration of BAIL inhibitors, a significant drop in both cathodic and anodic current densities occurred, indicating that both BAIL1 and BAIL2 could effectively suppress cathodic hydrogen evolution reactions and anodic metal dissolution. Moreover, the shift in the corrosion potential (*E*_corr_) towards more positive value corresponds to the dominant anodic inhibition of BAILs^31^. Inspection of the data of Table 2 reveals that the displacements of the corrosion potential in the presence of BAIL1 and BAIL2 are both less than 85 mV, which consequently suggests that BAILs act as mixed-type inhibitors^15^.

Furthermore, the values of *β*_c_ and *β*_a_ show only slight changes after the addition of BAIL1 and BAIL2, indicating that there is a minor effect on both cathodic hydrogen evolution and anodic iron dissolution, so the inhibition may mainly occur by a blocking mechanism due to the adsorption of BAIL inhibitors on the steel surface^32^.

The values of inhibition efficiency (*IE*_PDP_%) enhanced with the increase of the concentration of BAILs initially, reaching the maximum at the concentration of 0.75 mM, and then decreased slightly when further increasing concentration of BAILs. This can be explained as follows: BAILs are adsorbed onto the steel surface forming a protective film to block the active corrosion sites, thus retards the corrosion reaction; and the increase in concentration results in good inhibition efficiency (*IE*_PDP_%) by the increased coverage of BAIL adsorbed and/or the formation of corrosive products on the electrode surface^33^, which will be further confirmed by the subsequent XPS results. While extra inhibitor results in desorption of BAIL molecules from the electrode surface. The optimum inhibition efficiency (*IE*_PDP_%) of BAIL1 and BAIL2 in 0.5 M HCl solution is 81.6% and 89.5% at the concentration of 0.75 mM, respectively. These PDP results are in good harmony with those obtained from the EIS experiments.

### Weight loss measurements

The results obtained from weight loss experiments are listed in Table 3. At room temperature, the corrosion rate decreased first and then increased with the concentration of BAILs; while the inhibition efficiency(*IE*_WL_%) increased first and then decreased. The maximum inhibition efficiency (*IE*_WL_%) reaches 81.3% (BAIL1) and 89.7% (BAIL2) at the optimum concentration of 0.75 mM. These results obtained from the WL measurements are consistent with those from above EIS and PDP experiments.

**Table 3:** Corrosion parameters for carbon steel immersed in 0.5 M HCl solution for 24 h without/with different concentrations of BAIL1 and BAIL2 at 298 K obtained from WL measurements.

Inhibitor	*C*_inh_ (mM)	Δ*W* (mg)	*CR* (mg cm^−2^ h^−1^)	*IE*_WL_ (%)
blank	0	116.8	0.4167	
BAIL1	0.25	37.49	0.1337	67.9
	0.5	32.00	0.1142	72.6
	0.75	21.84	0.0779	81.3
	1.0	31.42	0.1121	73.1
BAIL2	0.25	25.46	0.0908	78.2
	0.5	23.24	0.0829	80.1
	0.75	12.03	0.0043	89.7
	1.0	21.61	0.0771	81.5

### SEM analysis

**Figure 6:**
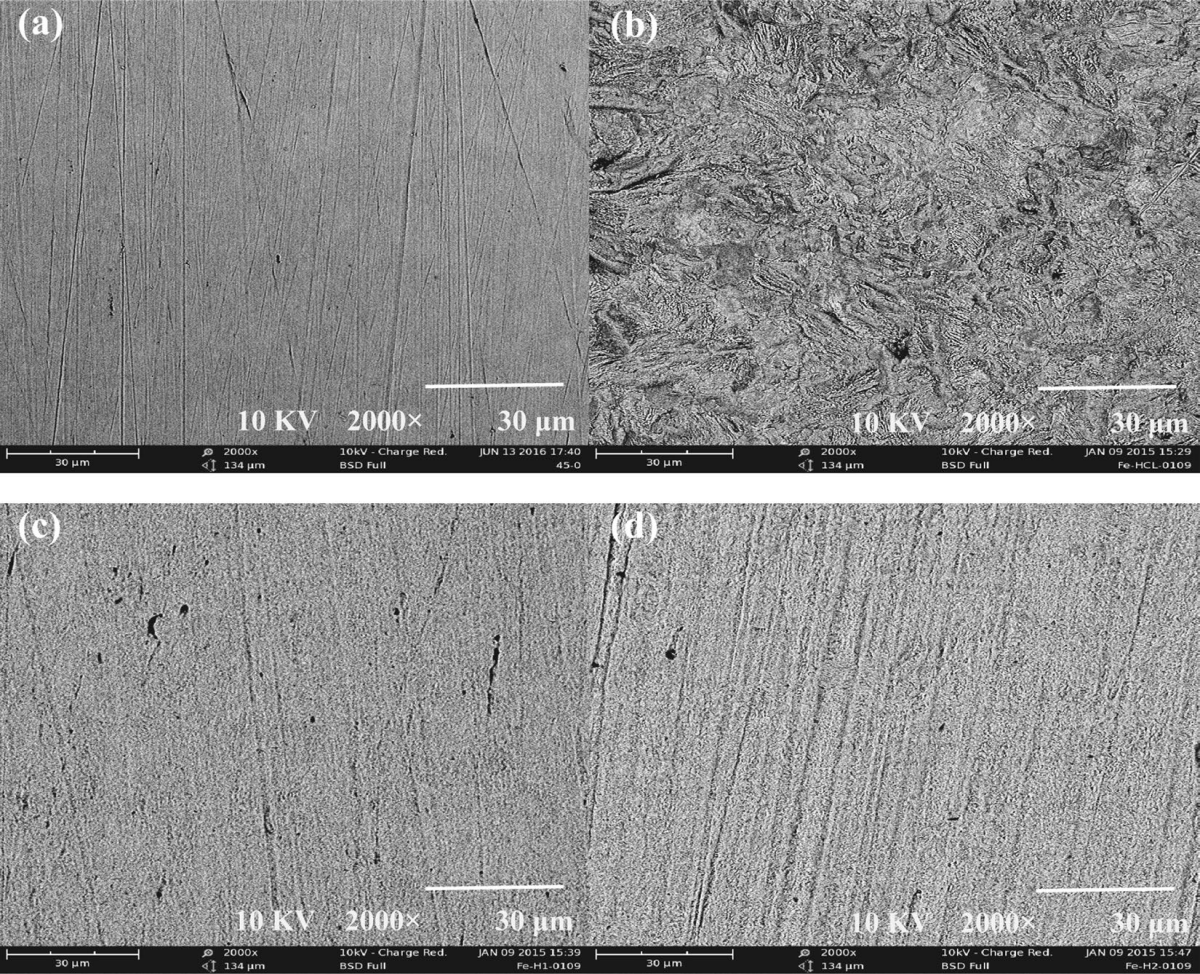
SEM micrographs of the steel surface with the magnification of 2000: fresh surface (**a**); after electrochemical tests in 0.5 M HCl solution (**b**); after electrochemical tests in 0.5 M HCl solution with 0.75 mM BAIL1 (**c**); after electrochemical tests in 0.5 M HCl solution with 0.75 mM BAIL2 (**d**).

SEM analysis is performed to study the corrosion morphology of the steel surface before and after electrochemical tests without or with 0.75 mM BAILs, and their SEM images are shown in Fig. 6.

The steel surface before electrochemical tests is uniform and only some abrading scratches can be observed in Fig. 6a, while seriously damaged surface is shown in uninhibited 0.5 M HCl solution (Fig. 6b); However, in the presence of the BAILs at optimum concentration (0.75 mM), the steel surface is smoother and less damaged, indicating good inhibition efficiency of the two BAILs (see Fig. 6c and d).

### XPS spectra analysis

**Figure 7:**
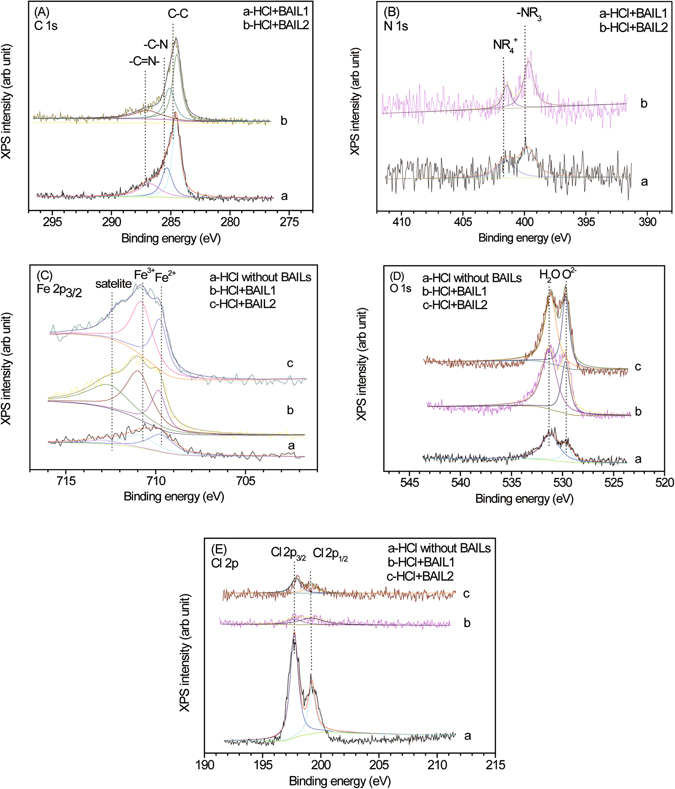
XPS spectra for carbon steel immersed in 0.5 M HCl solution without/with 0.75 mM BAILs after electrochemical experiments: (**A**) C 1 s; (**B**) N 1 s; (**C**) Fe 2p3/2; (**D**) O 1 s; (**E**) Cl 2p.

XPS spectra for C, N, Fe, O, Cl, S and P elements on carbon steel after PDP experiments in 0.5 M HCl solution in the absence and presence of 0.75 mM BAILs have been tested. In the C 1s XPS spectra, a peak at 284.6 eV can be assigned to the C-C bonds, and the other two can be ascribed to C-N (286.8 ± 0.2 eV) and C = N (285.4 ± 0.2 eV), respectively (Fig. 7A)^18^. In the N 1 s XPS spectra in Fig. 7B, a peak at 399.5 ± 0.2 eV can be attributed to NR_3_ and the signal at 401.5 ± 0.2 eV can be assigned to the positively charged nitrogen atom (NR_4_^+^) in the imizazolium^18^, which affirms the presence of BAIL on the steel surface. Meanwhile, S is not detectable on the steel surface inhibiting by BAIL1 in the spectrum of S 2p, while in the P 2p spectrum of BAIL2 inhibiting system, a small peak at around 133.3 eV could not be negligible and might be attributed to the corrosion product in the form of an insoluble phosphate, which may be one of the important reasons for the higher inhibiting efficiency of BAIL2 compared with BAIL1. The detailed spectra are included in the Supplementary Information (Fig. S7).

The corrosion products have been further studied by Fe, O and Cl spectra. The deconvoluted XPS spectra of Fe 2p3/2 in Fig. 7C shows three peaks, respectively. A peak at 709.6 ± 0.2 eV is assigned to ferrous compound, and the peak at 710.9 eV can be attributed to ferric compound, while a peak at 713 eV can be ascribed to the satellite peak of ferric compound^16, 34^. The corrosion products on the steel surface are obtained after BAILs inhibiting according to Fe XPS spectra, which can also retard the dissolution process of the steel electrodes together with the adsorption of BAILs.

In the spectra of O 1 s (Fig. 7D), a peak at 530.2 eV can be attributed to O^2−^ of ferric oxides such as Fe_2_O_3_ and/or Fe_3_O_4_, and the peak of OH^−^ of hydrous iron oxides (i.e., FeOOH) appears at 531.7 ± 0.2 eV^35^. In the spectra of Cl 2p in Fig. 7E, a peak at 198.7 ± 0.2 eV can be attributed to Cl 2p _3/2_, and the peak at 199.2 ± 0.2 eV can be ascribed to Cl 2p _1/2_^36^. The steel surface could be accessible by lots of Cl^−^ and H^+^ ions in the absence of corrosion inhibitors, which leads to serious corrosion, while in the presence of BAILs, less Cl^−^ ion appears on the steel surface, which can be explained that BAILs adsorbed on the steel surface block active sites on the steel surface against the attack of Cl^−^^33^.

Hence, in the XPS spectra, we can conclude that the adsorbed cation of BAILs on the steel surface and the decrease in Cl^−^ attack may lead to the efficient corrosion inhibition.

### Adsorption isotherms and thermodynamic characterization

**Figure 8:**
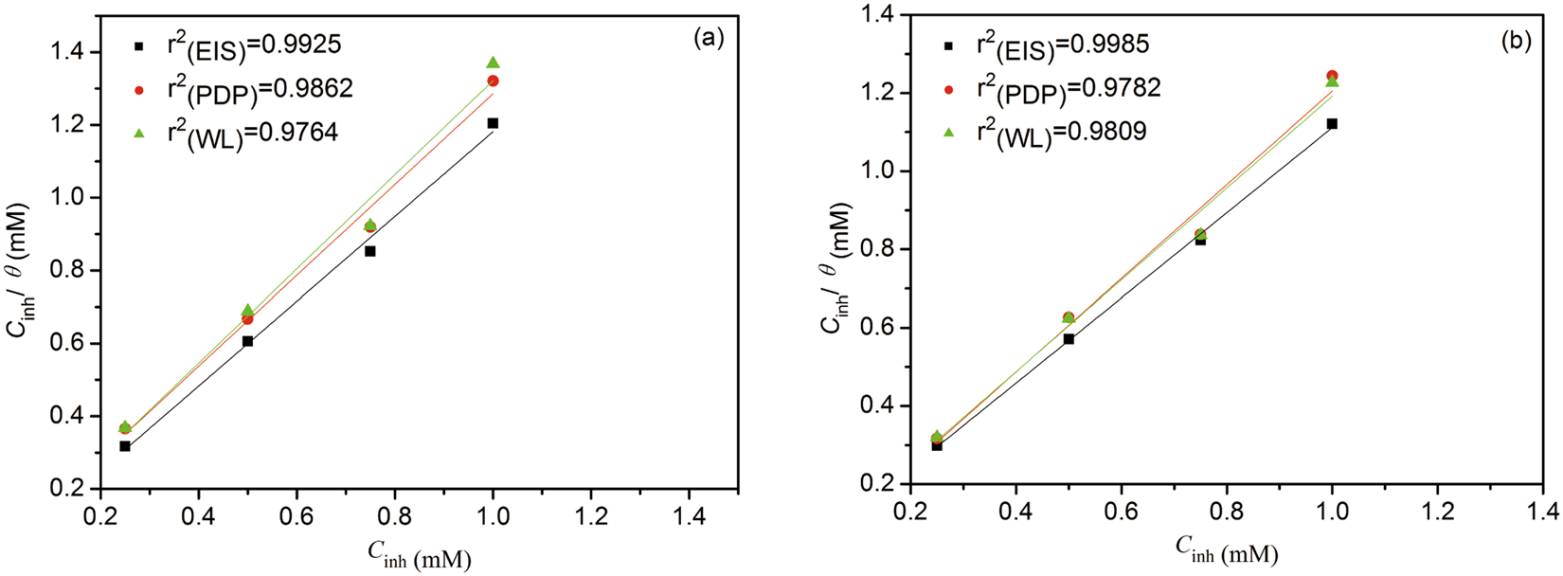
Langmuir isotherm for carbon steel in 0.5 M HCl solution with different concentrations of BAIL1 (**a**) and BAIL2 (**b**) calculated by EIS (), PDP () and WL () results.

To provide a better understanding of adsorption mechanism of BAILs on the steel surface, several adsorption isotherms including Temkin, Frumkin, Freundlich (see the Supplementary Information Figs S7–S9) and Langmuir adsorption isotherm are used to understand the interaction mechanism between BAILs and the steel surface. According to the above adsorption isotherms, the surface coverage (*θ*) is related to the concentration (*C*) of inhibitors according to the following equations:

8 $${\rm{L}}{\rm{a}}{\rm{n}}{\rm{g}}{\rm{m}}{\rm{u}}{\rm{i}}{\rm{r}}:\theta /(1-\theta )={K}_{{\rm{a}}{\rm{d}}{\rm{s}}}{C}_{{\rm{i}}{\rm{n}}{\rm{h}}}$$

9 $${\rm{Freundlich}}:\,\mathrm{log}\,\theta =\,\mathrm{log}\,{K}_{{\rm{ads}}}+1/n\,\mathrm{log}\,{C}_{{\rm{inh}}}$$

10 $${\rm{Temkin}}:\,\mathrm{log}(\theta /{C}_{{\rm{inh}}})=\,\mathrm{log}\,{K}_{{\rm{ads}}}-g\theta $$

11 $${\rm{Frumkin}}:\,\mathrm{log}(\theta /(1-\theta ){C}_{{\rm{inh}}})=\,\mathrm{log}\,{K}_{{\rm{ads}}}+g\theta $$

where *K*_ads_ is the adsorption equilibrium constant, *C*_inh_ is the inhibitor concentration, *g* is the adsorbate interaction parameter, *n* is constant at any temperature. The above adsorption isotherms were all tested in an attempt to fit the experimental data, however, only the Langmuir adsorption isotherm provided the best linear fits with the value of correlation coefficient (*R* ^2^) > 0.9, and was finally chosen for further study the mechanism of corrosion inhibition for BAILs. The intercept was applied to calculate the *K*_ads_, and the adsorption free energy (Δ*G* ^0^_ads_) was estimated from the follow equation.

12 $${\rm{\Delta }}{G0}_{{\rm{a}}{\rm{d}}{\rm{s}}}=-RT\,{\rm{l}}{\rm{n}}(55.5{K}_{{\rm{a}}{\rm{d}}{\rm{s}}})$$

where *R* is the universal gas constant and *T* is the absolute temperature. The adsorption of BAILs molecules on the steel surface in 0.5 M HCl solution obeys the Langmuir adsorption isotherm according to the plot of *C*_inh_/*θ* versus *C*_inh_^37, 38^ in Fig. 8, and the Δ*G* ^0^_ads_ obtained from Langmuir adsorption isotherm for the adsorption of BAIL1 and BAIL2 are provided in Table 4.

**Table 4:** Thermodynamic parameters for adsorption of BAIL1 and BAIL2 on the steel surface in 0.5 M HCl solution obtained from EIS, PDP and WL measurements.

Inhibitor	*K*_ads(EIS)_	*K*_ads(PDP)_	*K*_ads(WL)_	Δ*G* ^0^_ads(EIS)_	Δ*G* ^0^_ads(PDP)_	Δ*G* ^0^_ads(WL)_
				(KJ mol^−1^)	(KJ mol^−1^)	(KJ mol^−1^)
BAIL1	32.15	25.77	35.03	−18.55	−18.00	−18.76
BAIL2	52.08	121.95	54.64	−19.74	−21.85	−19.86

Generally, The negative value of free energy means that the adsorption process occurs spontaneously, and the values of adsorption free energy (Δ*G* ^0^_ads_) up to −20 kJ mol^−1^ or less negative usually correspond to physical adsorption, namely the electrostatic interaction between the charged metal and the charged inhibitor, while those more negative than −40 kJ mol^−1^ are assigned to chemisorption by sharing or transferring electrons from organic molecules to the metal surface to form a coordinate type of bond^39, 40^. Therefore, the interaction of BAIL inhibitors with the steel surface takes place mainly through physisorption, with Δ*G* ^0^_ads_ value between −18.00 and −21.85 kJ mol^−1^ shown in Table 4. However, chemisorption could not be completely excluded. As compared with BAIL1, the more negative value of Δ*G* ^0^_ads_ of BAIL2 and the higher value of the adsorption equilibrium constant *K*_ads_ in aqueous 0.5 M HCl with BAIL2 indicates that BAIL2 exhibits a slightly stronger tendency to be adsorbed on the steel surface, and consequently provides a higher corrosion inhibiting efficiency.

The effect of temperature in the range of 298–328 K on the corrosion rates of the steel was studied by weight loss method in 0.5 M HCl solution in the absence and presence of 0.75 mM of two BAILs for 24 h shown in Table 5. Regardless of the presence of the inhibitors, the corrosion rate increased with temperature. In the presence of BAIL, the inhibition efficiency (*IE*_WL_%) decreased with the increase of temperature, which was due to the fact that the metal dissolution process was enhanced and the adsorbed BAIL molecules were partially desorbed from the steel surface when increasing temperature^41^.

**Table 5:** Corrosion parameters for carbon steel immersed in 0.5 M HCl solution for 24 h without/with 0.75 mM BAIL1 and 0.75 mM BAIL2 at 298 K, 308 K, 318 K and 328 K obtained from WL measurements.

Inhibitor	*C*_inh_	298 K		308 K		318 K		328 K	
	mM	*CR* (mg cm^−2^ h^−1^)	*IE*_WL_ (%)	*CR* (mg cm^−2^ h^−1^)	*IE*_WL_ (%)	*CR* (mg cm^−2^ h^−1^)	*IE*_WL_ (%)	*CR* (mg cm^−2^ h^−1^)	*IE*_WL_ (%)
blank	0	0.4167		0.8490		1.7690		2.9671	
BAIL1	0.75	0.0779	81.3	0.1816	78.69	0.5435	69.3	1.2658	57.3
BAIL2	0.75	0.0430	89.7	0.1175	86.2	0.3465	80.4	0.8406	71.7

**Figure 9:**
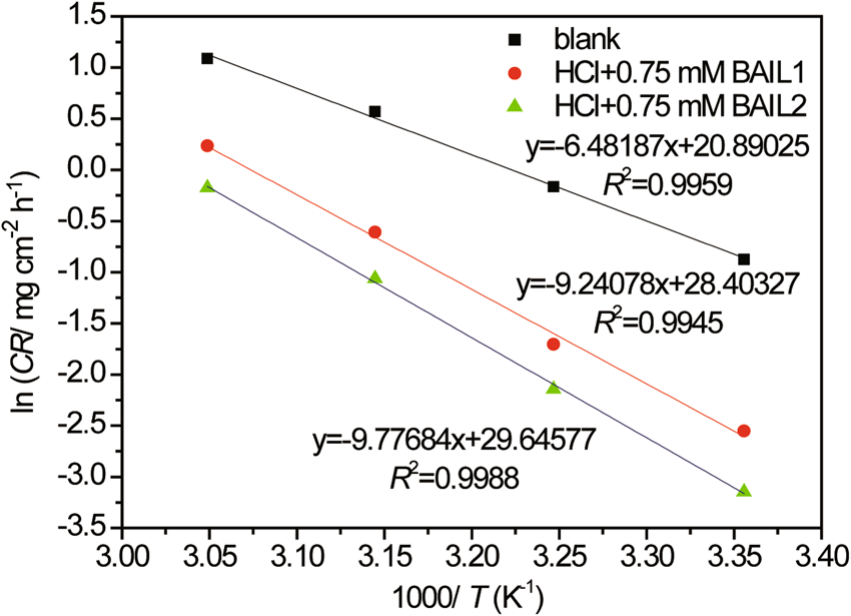
Arrhenius plot for carbon steel in 0.5 M HCl solution without BAIL () and with 0.75 mM of BAIL1 () and BAIL2 ().

The apparent activation energy (*E*_a_) was deduced using the following equation:

13 $$\mathrm{ln}(CR)=-{E}_{{\rm{a}}}/RT+A$$

where *CR* is the corrosion rate, *E*_a_ is the apparent activation energy, *R* is the general gas constant, *T* is the absolute temperature and *A* the Arrhenius pre-exponential factor. A plot of log*CR* of carbon steel obtained from weight loss experiments vs. 1000/*T* gave straight lines as shown in Fig. 9. The values of *E*_a_ determined from the slope of the lines are given in Table 6.

**Table 6:** The values of activation parameters *E*_a_, Δ*H* ^0^_a_ and Δ*S* ^0^_a_ for carbon steel in 0.5 M HCl solution without/with 0.75 mM BAIL1 and 0.75 mM BAIL2 obtained from WL measurements.

Inhibitor	*C*_inh_ (mM)	*E*_a_ (KJ mol^−1^)	Δ*H* ^0^_a_ (KJ mol^−1^)	Δ*S* ^0^_a_ (J mol^−1^ K^−1^)
Blank	0	53.9	51.3	−80.2
BAIL1	0.75	76.8	74.2	−17.4
BAIL2	0.75	81.3	78.7	−7.2

**Figure 10:**
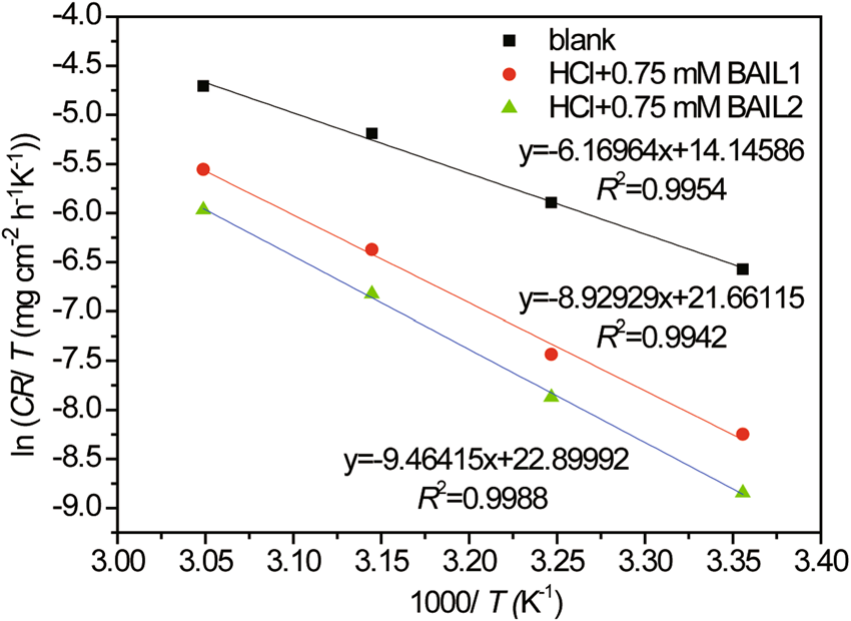
Plot of ln(*CR*/*T*) vs. 1000/T for carbon steel in 0.5 M HCl solution without BAILs () and with 0.75 mM of BAIL1 () and BAIL2 ().

Δ*S* ^0^_a_ and Δ*H* ^0^_a_ were calculated using an alternative formula of the Arrhenius equation^40^:

14 $$CR=RT/Nh\exp \,({\rm{\Delta }}{S}_{{\rm{a}}}^{0}/R)\exp (-{\rm{\Delta }}{H}_{{\rm{a}}}^{0}/RT)$$

where *h* is the Planck’s constant, *N* is the Avogadro’s number, Δ*S* ^0^_a_ is the activation entropy and Δ*H* ^0^_ads_ is the activation enthalpy. Figure 10 shows a plot of ln(*CR*/*T*) vs. 1000/*T*, from which the values of Δ*S* ^0^_a_ and Δ*H* ^0^_a_ are calculated, and also listed in Table 6.

Generally, increased activation energy (*E*_a_) indicates physical adsorption which occurs in the first stage or weak chemical bonding between the inhibitors and the steel surface, while unchanged or lower *E*_a_ in the presence of inhibitor suggests chemisorption^42, 43^. It is seen from Table 6 that *E*_a_ and Δ*H* ^0^_a_ values are higher in inhibited solution compared to those in the blank, which shows that the energy barrier of corrosion reaction increases, and also supports that physisorption of inhibitors occurs dominantly. The value of Δ*S* ^0^_a_ is less negative in the presence of BAILs than that without BAILs, and the increase of Δ*S* ^0^_a_ is generally interpreted by the increase in disorder taking place in going from reactants to the activated complex^44, 45^.

### UV–visible spectrophotometric studies

**Figure 11:**
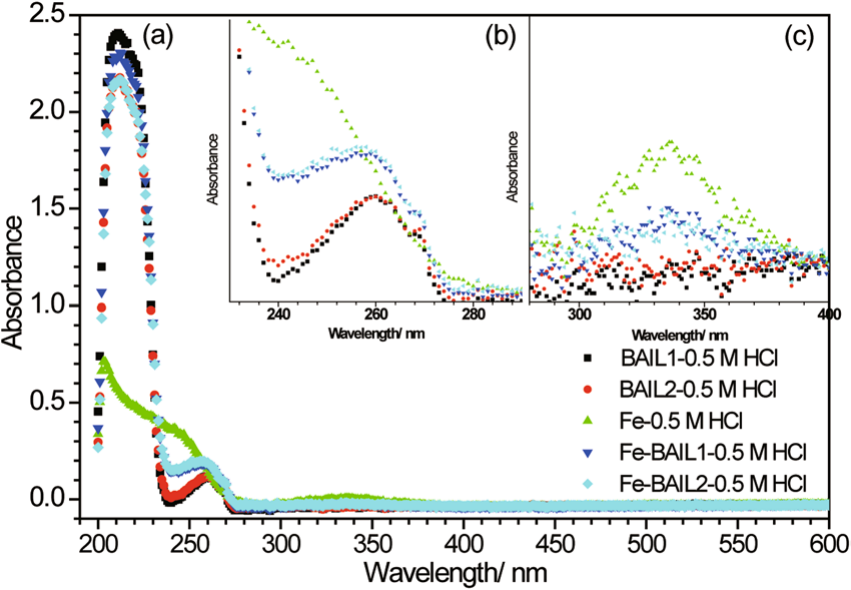
UV–visible spectra of 0.5 M HCl solution without/with 0.75 mM BAIL1 and BAIL2 before and after steel immersion of 24 h. Note: 0.5 M HCl solution +BAIL1 (); 0.5 M HCl solution +BAIL2 (); 0.5 M HCl solution after steel immersion (); 0.5 M HCl solution +BAIL1 after steel immersion (); 0.5 M HCl solution +BAIL2 after steel immersion ().

UV–visible spectroscopic measurements were undertaken for 0.5 M HCl solution without or with 0.75 mM BAIL1 or BAIL2 before and after 24 h immersion of carbon steel at room temperature, since the position of absorbance maximum and the peak intensity will change suggesting the formation of a complex between two species in solution^46, 47^. The spectra are illustrated in Fig. 11.

In the presence of BAILs before or after steel immersion, all the optical spectra show two broad bands, one is strong in the range of 200–230 nm and the other is moderate-intensity at around 258 nm. The first band is attributed to the π → π* transition of phenyl or imidazole ring and n → π* electronic transition from carboxyl groups; The latter could be mainly assigned as π → π* electronic transition related to aromatic ring^46^, however, the contribution of n → π* electronic transition from carboxyl group could not be excluded. The enhancement in peak intensity at 258 nm in inhibited solution of BAIL1/BAIL2 means the formation of complex between Fe^2+^ released and the BAILs in solution. A weak absorption peak at around 336 nm is related to the interaction between Fe^2+^ and chloride complex^47^, and the peak intensity in the absence of inhibitors is stronger than that in the inhibited solution, which demonstrates the corrosion inhibition capability of BAIL1 and BAIL2.

### Postulated mechanism of inhibition

Organic inhibitors generally prevent the metal from corrosion by adsorption on the metal surface. Here the adsorption of BAILs is primarily a physisorption process which obeys Langmuir adsorption isotherm best due to electrostatic interaction, although weak chemisorption could not be excluded, which can be confirmed by the UV–visible spectroscopic studies.

The adsorption process not only depends on the kind of inhibitors, also depends on the surface charge of metal employed and the aggressive solution used^48^. According to previous references, the surface of carbon steel in acidic solution is positively charged at its OCP in various concentrations of HCl solution^48, 49^. This situation will firstly favor the adsorption of the negatively charged ions such as Cl^−^ onto the surface of carbon steel, if considering the electrostatic attraction. Therefore, according to the above discussion, the possible inhibition mechanism is predicted.

The anodic reaction (Fe dissolution) of carbon steel in 0.5 M HCl solution may be given as follows^50^:

$$\begin{array}{c}{\rm{F}}{\rm{e}}+{{\rm{C}}{\rm{l}}}^{-}({\rm{a}})\leftarrow \to {({{\rm{F}}{\rm{e}}{\rm{C}}{\rm{l}}}^{-})}_{{\rm{a}}{\rm{d}}{\rm{s}}}({\rm{b}})\leftarrow \to ({{\rm{F}}{\rm{e}}{\rm{C}}{\rm{l}}}^{-}{)}_{{\rm{a}}{\rm{d}}{\rm{s}}}+{{\rm{e}}}^{-}({\rm{c}})\to {{\rm{F}}{\rm{e}}{\rm{C}}{\rm{l}}}^{+}+{{\rm{e}}}^{-}({\rm{d}})\leftarrow \to {{\rm{F}}{\rm{e}}}^{2+}+{{\rm{C}}{\rm{l}}}^{-}({\rm{e}})\\ \quad \quad \quad \quad \quad \quad \quad \quad \Updownarrow \\ 2{({{\rm{F}}{\rm{e}}{\rm{C}}{\rm{l}}}^{-})}_{{\rm{a}}{\rm{d}}{\rm{s}}}({\rm{b}})+{{\rm{B}}{\rm{A}}{\rm{I}}{\rm{L}}}^{2+}\to {[{\rm{F}}{\rm{e}}(2{{\rm{C}}{\rm{l}}}^{-}){{\rm{B}}{\rm{A}}{\rm{I}}{\rm{L}}}^{2+}]}_{{\rm{a}}{\rm{d}}{\rm{s}}}({\rm{f}})\leftarrow \to {({{\rm{F}}{\rm{e}}{\rm{B}}{\rm{A}}{\rm{I}}{\rm{L}}}^{2+})}_{{\rm{a}}{\rm{d}}{\rm{s}}}+2{{\rm{C}}{\rm{l}}}^{-}({\rm{g}})\end{array}$$

Due to the concentration of Cl^−^ is much higher than HSO_4_^−^ and H_2_PO_4_^−^ (the anion of BAIL1 and BAIL2), the adsorption and desorption of HSO_4_^−^/H_2_PO_4_^−^ can be neglected. In the absence of BAIL inhibitors, the mechanism of the anodic dissolution reactions follows the pathway (a→b→c→d→e)^51^. However, in the presence of BAIL inhibitors, the reaction pathway (a→b→f or a→b→f→g) is favored. Thus, the Cl^−^ is also blocked by the cation of BAIL inhibitors.

The cathodic reactions (hydrogen evolution) of carbon steel in 0.5 M HCl solution are given as follows:

$$\begin{array}{c}{\rm{Fe}}+{{\rm{H}}}^{+}({\rm{a}}\mbox{'})\leftarrow \to {({{\rm{FeH}}}^{+})}_{{\rm{ads}}}+{{\rm{e}}}^{-}({\rm{b}}\mbox{'})\to {({\rm{FeH}})}_{{\rm{ads}}}+{{\rm{H}}}^{+}+{{\rm{e}}}^{-}({\rm{c}}\mbox{'})\to {\rm{Fe}}+{{\rm{H}}}_{2}({\rm{d}}\mbox{'})\\ \updownarrow \,+{{\rm{BAIL}}}^{2+}\\ {({{\rm{FeBAIL}}}^{2+})}_{{\rm{ads}}}({\rm{e}}\mbox{'})\\ \downarrow +2{{\rm{e}}}^{-}\\ {({\rm{FeBAIL}})}_{{\rm{ads}}}({\rm{f}}\,\mbox{'})\end{array}$$

In the absence of BAIL inhibitors, the reaction pathway seems to be through the pathway (a’→b’→c’→d’). Whereas in the presence of BAIL inhibitors, the cation of BAIL can also be adsorbed at cathodic sites in competition with hydrogen ions that going to reduce H_2_ gas evolution. So the reaction pathway (a’→e’→f’) is favored.

In addition, because of the weak chemisorption exists between the BAILs and steel surface, the adsorption of the cation of BAILs shows a stronger preference over the competitive adsorption of H^+^. Meanwhile, the phenyl and imidazolium rings can cover a large part of the steel surface against further corrosion^33^. Therefore, good corrosion inhibition capacity could be obtained.

No great difference in inhibition efficiency of the two BAILs could be explained that the two BAILs have the same cation, which contributes much on the inhibiting capability; However, the effect of anion cannot be negligible due to the fact that the corrosion inhibiting efficiency of BAIL2 is slightly better than that of BAIL1. This may be caused by the higher acidity of HSO_4_^−^ than that of H_2_PO_4_^−^ in their aqueous solution, meanwhile, the possible corrosion product of ferric phosphate is more insoluble than ferric sulfate^52, 53^, and could form a protective film on the steel surface, which has been demonstrated by XPS result that a small peak at 133.3 eV could be attributed to the corrosion product as an insoluble phosphate.

## Conclusions

Two Brönsted acid ionic liquids have been synthesized and acted as effective and green corrosion inhibitors for carbon steel in 0.5 M HCl solution, suggesting that the two BAILs can work as acidic catalyst and corrosion inhibitors in acidic medium simultaneously, and this will greatly speed up the industrial use of BAILs.

1. The PDP studies showed that the two BAILs acted as mixed-type inhibitors and could effectively suppress both cathodic and anodic reactions. The results obtained from EIS, PDP and WL tests are in good agreement, the best inhibition efficiency (*IE*_EIS_%) of BAIL1 and BAIL2 can reach 88.0% and 91.1%, respectively.
2. The steel surface analyses from SEM and XPS demonstrate the effectiveness of inhibition, and the adsorption of BAILs cation on the steel surface contributes much to corrosion inhibition.
3. The adsorption of BAIL inhibitors on the steel surface was found to obey the Langmuir adsorption isotherm best. The values of activation energy and free energy of adsorption indicate that the physisorption of BAILs through electrostatic interaction is dominant.
4. UV-visible spectroscopic analysis illustrates the possible formation of the complex between BAIL inhibitors and Fe^2+^ released during the corrosion process, so that weak chemisorption cannot be excluded.

## Electronic supplementary material

Supporting information
