# Supplementary material for: Green Brönsted acid ionic liquids as novel corrosion inhibitors for carbon steel in acidic medium
Source: Sci Rep. 2017 Aug 18;7:8773. doi: 10.1038/s41598-017-07925-y (PMC5562793; doi:10.1038/s41598-017-07925-y)
Supplement: Supplementary file 1 — Supporting information [file 41598_2017_7925_MOESM1_ESM.pdf]

SUPPLEMENTARY INFORMATION

Green Brönsted acid ionic liquids as novel corrosion inhibitors for carbon steel in acidic medium

Shuyun Cao[a], Dan Liu[b*], Peng zhang[a], Lixia Yang[a], Peng Yang[c], Hui Lu[d], Jianzhou Gui[a,b*]

[a] *State Key Laboratory of Separation Membranes and Membrane Processes & School of Material Science and Engineering, Tianjin Polytechnic University, Tianjin, 300387, P.R. China*

[b] *School of Environmental and Chemical Engineering, Tianjin Polytechnic University, Tianjin, 300387, P.R. China*

[c] *Key laboratory of structure-Based Drug Design and Discovery of Ministry Education, Shenyang Pharmaceutical University, Shenyang, 110016, P.R. China*

[d] *State Key Laboratory of Catalysis, Dalian Institute of Chemical Physics, Chinese Academy of Sciences, Dalian, 116023, P.R. China*

*Corresponding author e-mail: *E-mail: danliu_939@hotmail.com; E-mail: jzgui@hotmail.com.*

Table of contents

Title of the paper, author's names, address	S1
Table of contents	S2
Materials	S3
Synthesis of ethyl 1H-imidazole-1-acetate (Compound 1)	S3
Synthesis of 1, 1'-(1, 4-phenylenebis(methylene))bis(3-(2-ethoxy-2-oxoethyl)-1H -imidazol-3-ium) bromide (Compound 2) and 1, 1'-(1, 4-phenylenebis(methylene)) bis(3-(carboxymethyl)-1H-imidazol-3-ium) chloride (Compound 3)	S4
Synthesis of BAILs 1, 1'-(1, 4-phenylenebis(methylene)) bis(3-(carboxymethyl)-1H-imidazol-3-ium) bisulfate (BAIL1) and dihydrogen phosphate (BAIL2)	S5
^{1}H NMR of BAIL1	S6
^{13}C NMR of BAIL1	S7
FTIR of BAIL1	S9
^{1}H NMR of BAIL2	S10
^{13}C NMR of BAIL2	S11
FTIR of BAIL2	S12
Supplementary X-ray photoelectron spectroscopy analysis	S13
Several adsorption isotherms including Temkin, Frumkin and Freundlich isotherm provided poor data fitting	S14

Number of pages: 16; Number of Figures: 10.

Experimental

Materials:

All solvents and chemicals in the experiments were analytical pure and used without further purification unless otherwise stated.

Synthesis of ethyl 1H-imidazole-1-acetate (Compound 1):

Prior to use, chloroform was pretreated by anhydrous sodium sulfate in order to remove water. A mixture of imidazole (6.808 g, 0.1 M), potassium carbonate (15.203 g, 0.110 M) in chloroform (50 mL) was heated to boiling, then added ethyl chloroacetate (12.255 g, 0.1 M) dropwise under stirring, and the mixture was refluxed at 75°C for 48 h till the reaction was completed. The mixture was then filtered and washed by another 50 mL chloroform to get the filtrate. After adding suitable amount of silica gel, the solvent was removed under reduced pressure. The mixture obtained was purified by column chromatography on silica gel (eluent: $CHCl_3$/ MeOH= 9/ 1) to give the second component (Compound 1) in 92% yield as a light yellow powder. The scheme of this reaction is as follows:

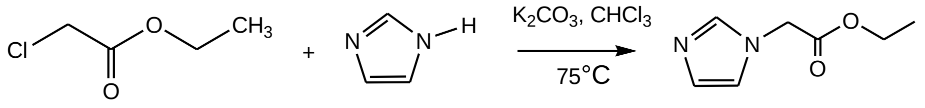

Compound 1

^{1}H-NMR (Compound 1) (600 MHz, DMSO-d_6, TMS): δ: 7.64(*s*, 1H), 7.16(*s*, 1H), 6.92(*s*, 1H), 4.97(*s*, 2H), 4.18~4.15(*m*, 2H), 1.24(*t*, *J*= 14.4 Hz, 3H).

^{13}C-NMR (Compound 1) (150 MHz, DMSO-d_6, TMS): δ: 168.99, 138.65, 128.51, 121.10, 61.56, 47.64, 14.35.

Anal. Calc. for $C_7H_{10}N_2O_2$: C, 54.54; H, 6.54; N, 18.17; O, 20.76. Found: C, 54.50; H, 6.57; N, 18.19; O, 20.70.

FTIR (Compound 1) (cm^{-1}): 3117, 2985, 2941, 1749, 1510, 1427, 1379, 1348, 873, 757.

Synthesis of 1, 1'-(1, 4-phenylenebis(methylene))bis(3-(2-ethoxy-2-oxoethyl)-1H -imidazol-3-ium) bromide (Compound 2) and 1, 1'-(1,4-phenylenebis(methylene)) bis(3-(carboxymethyl)-1H-imidazol-3-ium) chloride (Compound 3):

Synthesis of Compound 2: A mixture of 1.3-Bis(bromomethyl) benzene (13.198 g, 0.05 M) and ethyl imidazole acetate (16.959 g, 0.11 M) in acetonitrile (100 mL) was refluxed at 85°C for 48 h. After reaction, acetonitrile was removed under reduced pressure. Then the product obtained was dried under vacuum at 80℃ to give Compound 2 (in 94.0% yield). The scheme of the reaction is as follows:

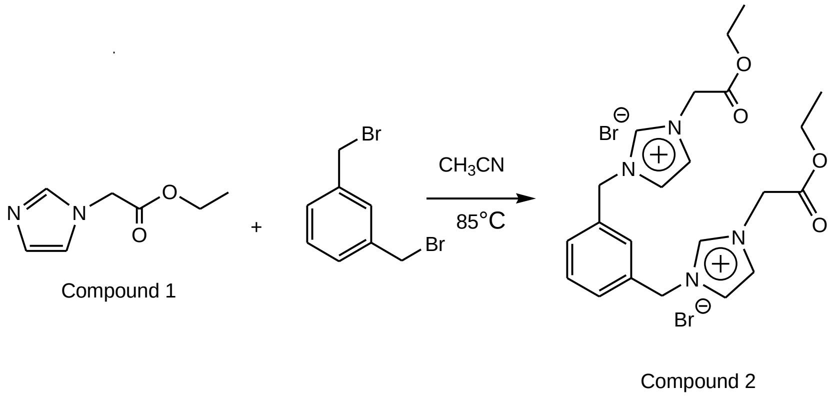

Synthesis of Compound 3: The obtained Compound 2 was dissolved in 1 M aqueous HCl solution (150 mL) and then refluxed at 110°C for 48 h. The solvent was evaporated under reduced pressure and then washed with acetone for 3 times, and finally dried under vacuum to obtain Compound 3 in 96.0% yield as a white powder. The scheme of the reaction is as follows:

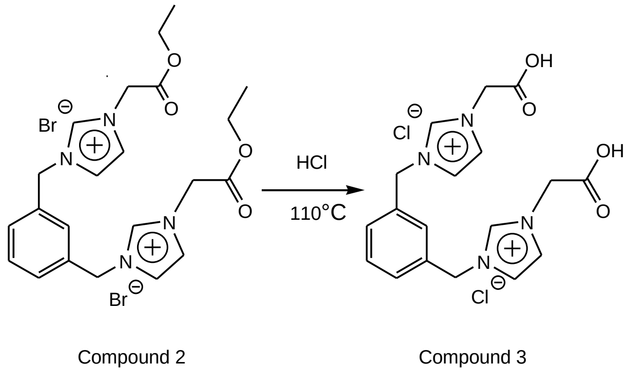

^{1}H-NMR (Compound 3) (600 MHz, DMSO-d_6, TMS): δ: 9.52(*s*, 2H), 7.95(*t*, *J*= 3.6 Hz, 2H), 7.84(*t*, *J*= 3.6 Hz, 2H), 7.63(*s*, 1H), 7.49~7.45(*m*, 3H), 5.58(*s*, 4H), 5.25(*s*, 4H).

^{13}C-NMR (Compound 3) (150 MHz, DMSO-d_6, TMS): 168.56, 137.90, 136.08, 130.26, 129.11, 128.85, 124.70, 122.60, 52.08, 50.39.

Anal. calc for $C_{18}H_{20}Cl_2N_4O_4$: C 50.60, H 4.72, N 13.11, O 14.98; Found: C 50.58, H 4.71, N 13.12, O 15.50.

FTIR (Compound 3) (KBr) (cm^{-1}): 3163, 3099, 2933, 1733, 1595, 1567, 1428, 1350, 874, 750.

Synthesis of BAILs 1, 1'-(1, 4-phenylenebis(methylene)) bis(3-(carboxymethyl)-1H-imidazol-3-ium) bisulfate (BAIL1) and dihydrogen phosphate (BAIL2):

A mixture of Compound 3 (4.273 g, 0.01 M) and concentrated sulfuric acid (2.002 g, 0.02 mM) or concentrated phosphoric acid (2.306 g, 0.02 mM) in dichloromethane (50 mL) were refluxed at 40°C for 60 h under stirring. The concentrated sulfuric acid/ concentrated phosphoric acid was added dropwise. When the reaction was finished, the dichloromethane was removed under reduced pressure, and the mixture obtained was washed by diethyl ether, then dried under vacuum to give the final target as yellow liquid in 99.0% (BAIL1)/ 98.0% (BAIL2) yield, respectively. The scheme of the reaction is as follows:

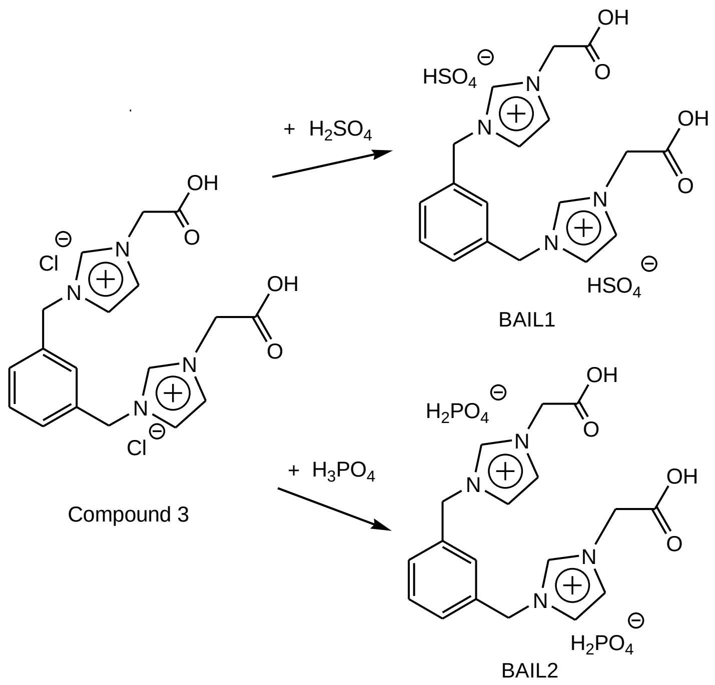

^{1}**H-NMR** (BAIL1) (DMSO-d_6, 600 MHz): δ: 9.39(*d*, *J*= 9.0 Hz, 2H, NCHN), 7.89(*d*, *J*= 12.6 Hz, 2H, $CH_3NCHCHN$), 7.78(*d*, *J*= 8.4 Hz, 2H, $CH_3NCHCHN$), 7.64(*s*, 1H, Ar-H), 7.46~7.42(*m*, 3H, Ar-H), 5.50(*s*, 4H), 5.17(*s*, 4H, NCH_2).

^{13}**C NMR** (BAIL1) (DMSO-d_6, 150 MHz): δ: 168.18, 137.92, 136.07, 130.27, 129.16, 128.98, 124.76, 122.60, 52.13, 50.30.

Anal. Calc. for $C_{18}H_{22}N_4O_{12}S_2$: C 39.27, H 4.03, N 10.18, O 34.88, S 11.65; Found: C 39.25, H 4.10, N 10.20, O 34.81, S 11.64.

FTIR (BAIL1) (KBr) (cm^{-1}): 3400, 3162, 3099, 3000, 2953, 2682, 2591, 2490, 1736, 1630, 1567, 1444, 1346, 1000–650.

^{1}**H-NMR** (BAIL2) (DMSO-d_6, 600 MHz): δ: 9.44(*s*, 2H, NCHN), 7.87(*s*, 2H, $CH_3NCHCHN$), 7.78(*s*, 2H, $CH_3NCHCHN$), 7.60(*s*, 1H, Ar-H), 7.44(*s*, 3H, Ar-H), 5.54(*s*, 4H), 5.17(*s*, 4H, NCH_2).

^{13}**C NMR** (BAIL2) (DMSO-d_6, 150 MHz): δ: 168.71, 137.79, 136.07, 130.26, 129.13, 128.75, 124.68, 122.50, 52.06, 50.64.

Anal. Calc. for $C_{18}H_{24}N_4O_{12}P_2$: C 39.13, H 4.38, N 10.14, O 34.75; Found: C 39.09, H 4.40, N 10.10, O 34.81.

FTIR (BAIL2) (KBr) (cm^{-1}): 3400, 3146, 3086, 2982, 2950, 2582, 2496, 1741, 1631, 1565, 1450, 1367, 1000–650.

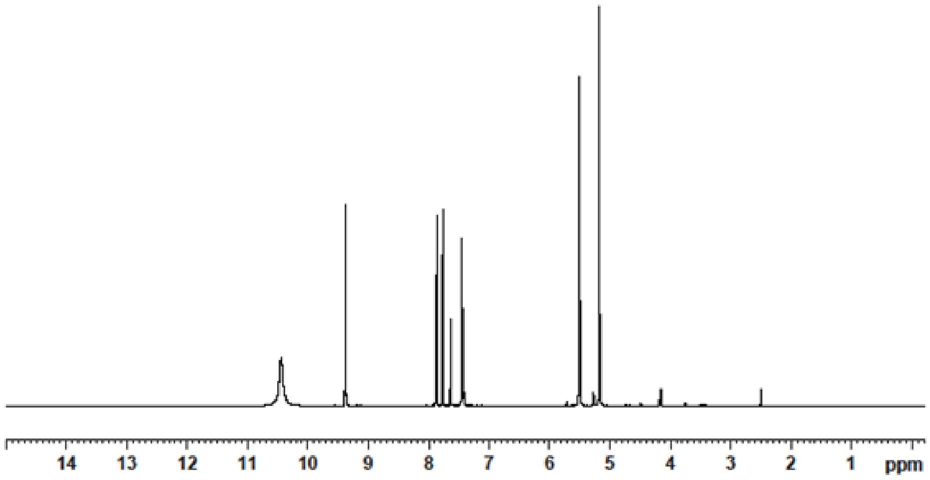

Fig. S1 ^{1}H NMR of BAIL1

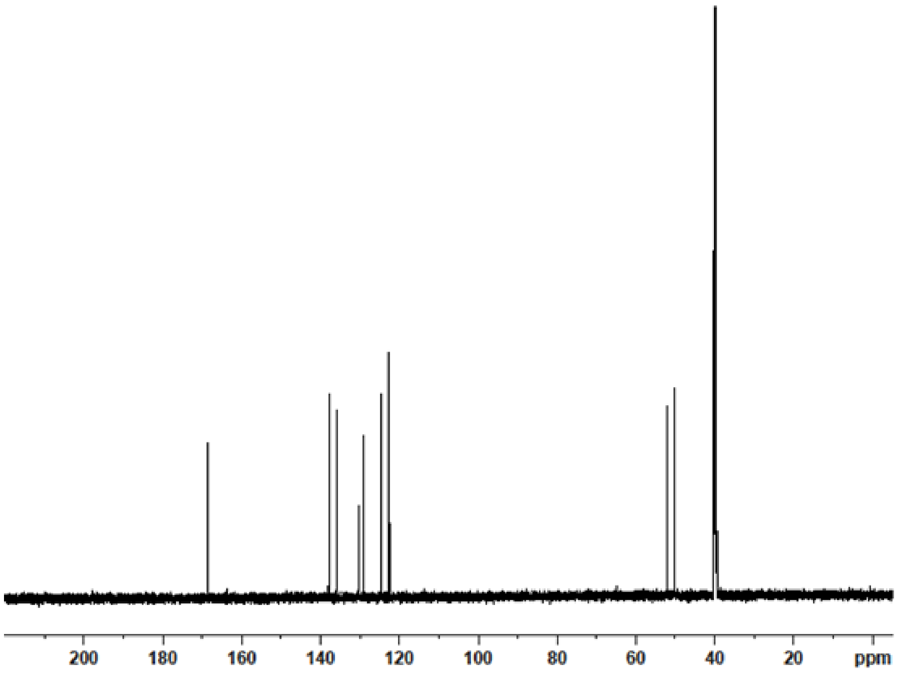

Fig. S2 ^{13}C NMR of BAIL1

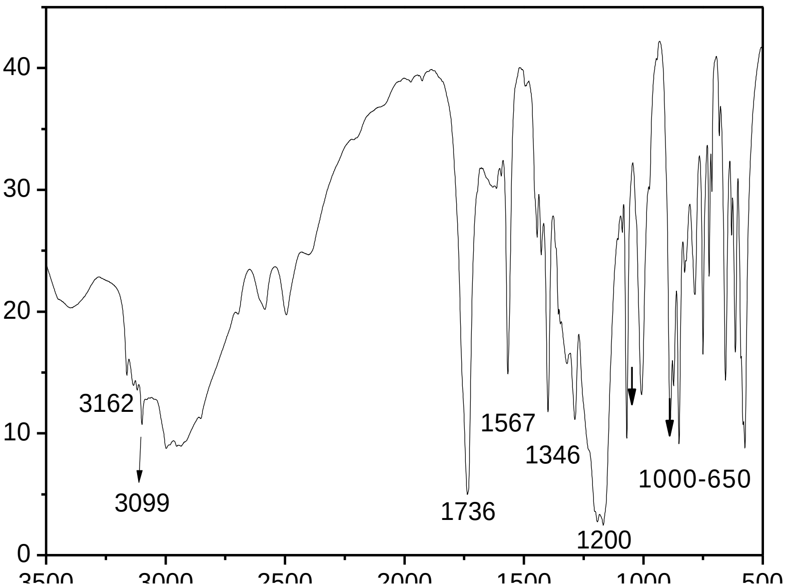

Fig. S3 FTIR of BAIL1

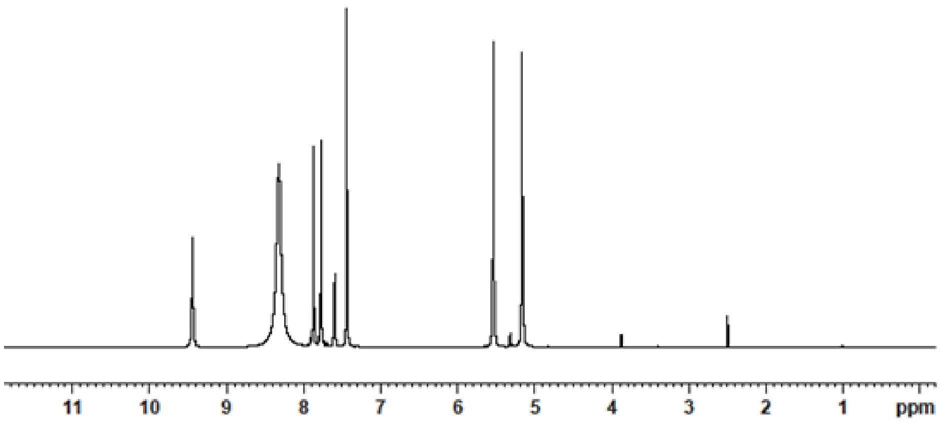

Fig. S4 ^{1}H NMR of BAIL2

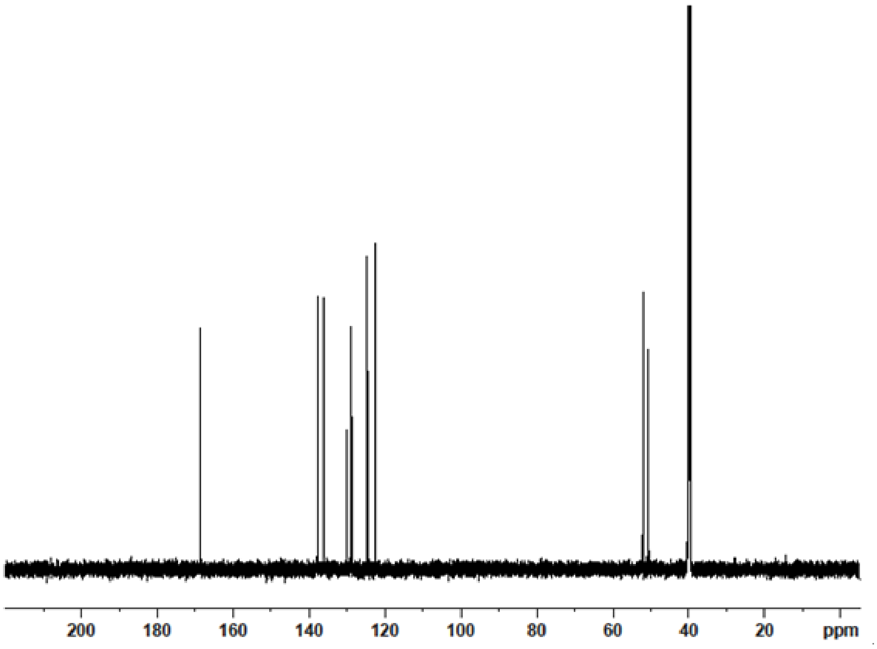

Fig. S5 ^{13}C NMR of BAIL2

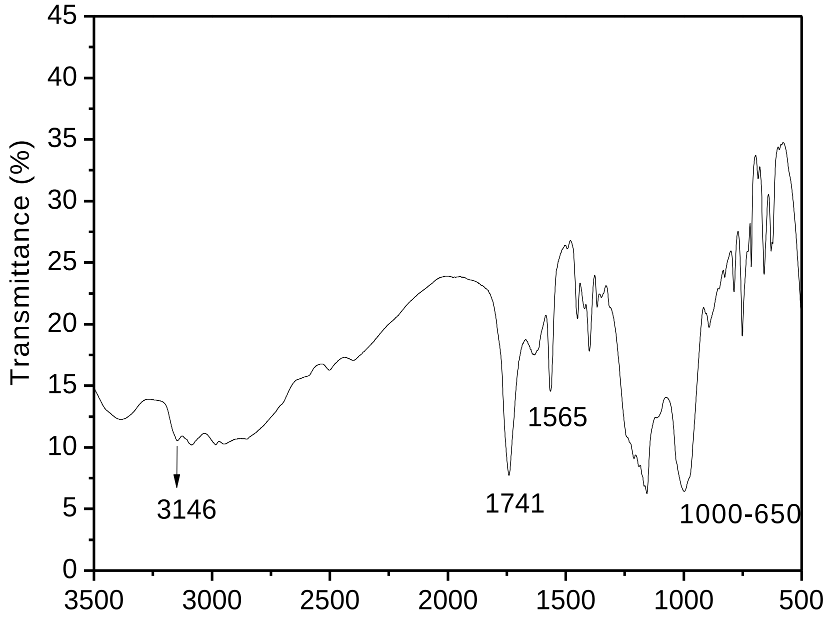

Fig. S6 FTIR of BAIL2

Supplementary X-ray photoelectron spectroscopy analysis:

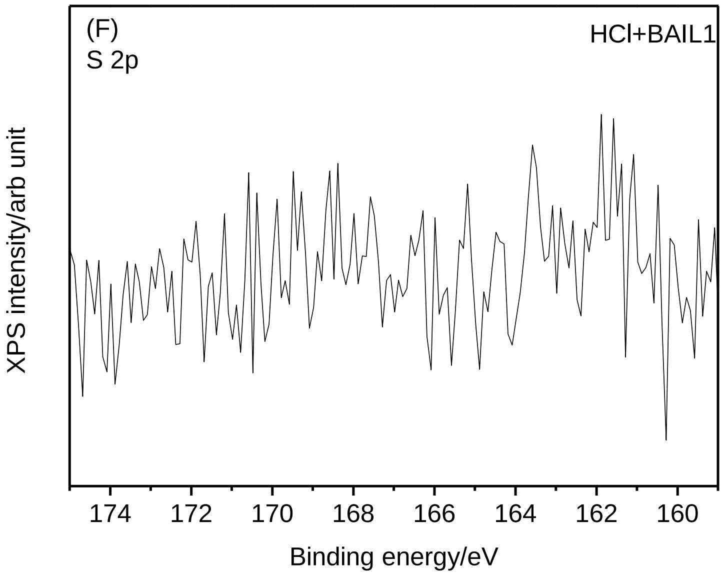

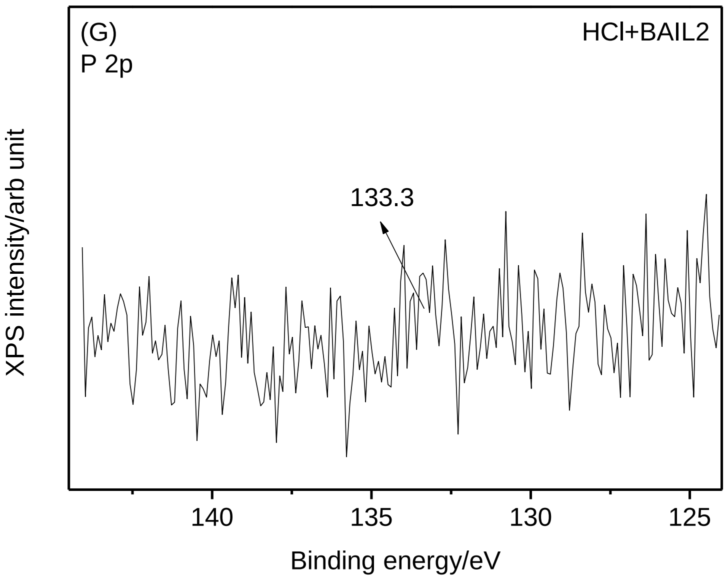

Fig. S7 XPS spectra for carbon steel immersed in 0.5 M HCl solution when inhibited by 0.75 mM BAILs: (F) S 2p spectrum in the presence of BAIL1 and (G) P 2p spectrum in the presence of BAIL2

Several adsorption isotherms including Temkin (see Fig. S7), Frumkin (see Fig. S8) and Freundlich isotherm (see Fig. S9) provided poor data fitting, shown as follows:

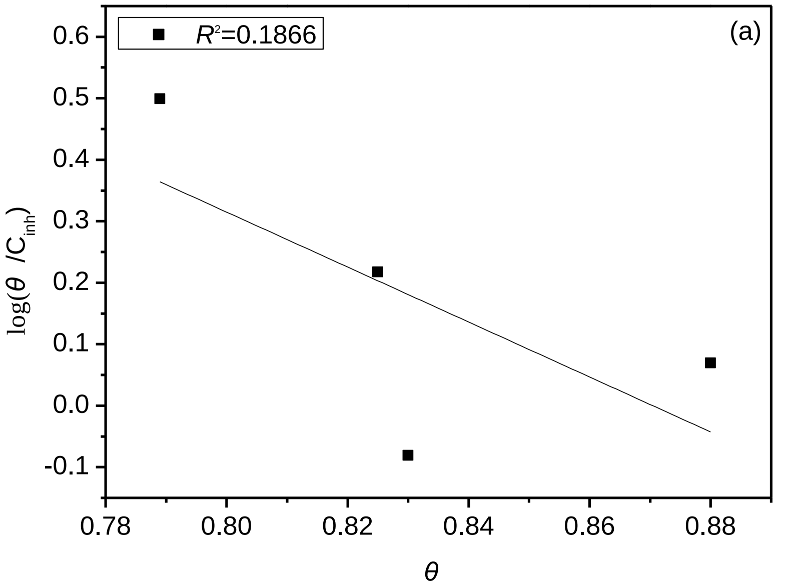

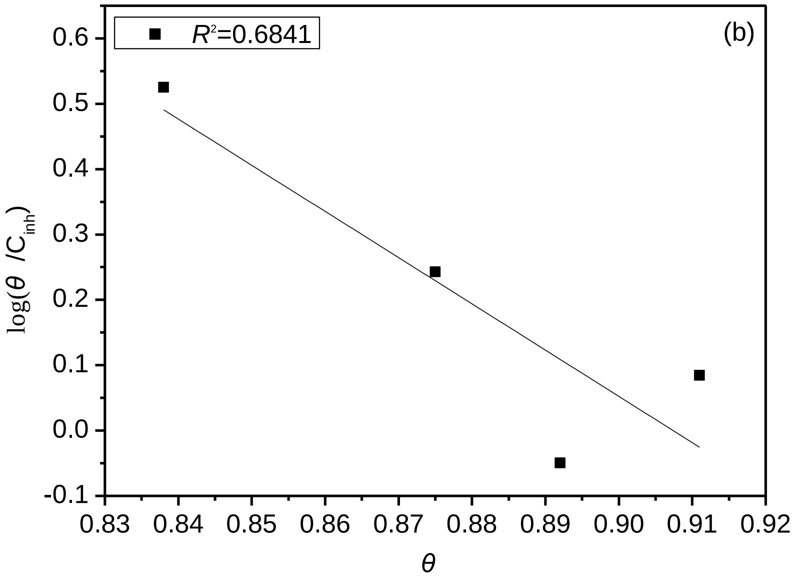

Fig. S8 Temkin isotherm (log (θ/ C_{inh}) = log K_{ads} – $g\theta$ for carbon steel in 0.5 M HCl solution with different concentrations of BAIL1 (a) and BAIL2 (b) using EIS data

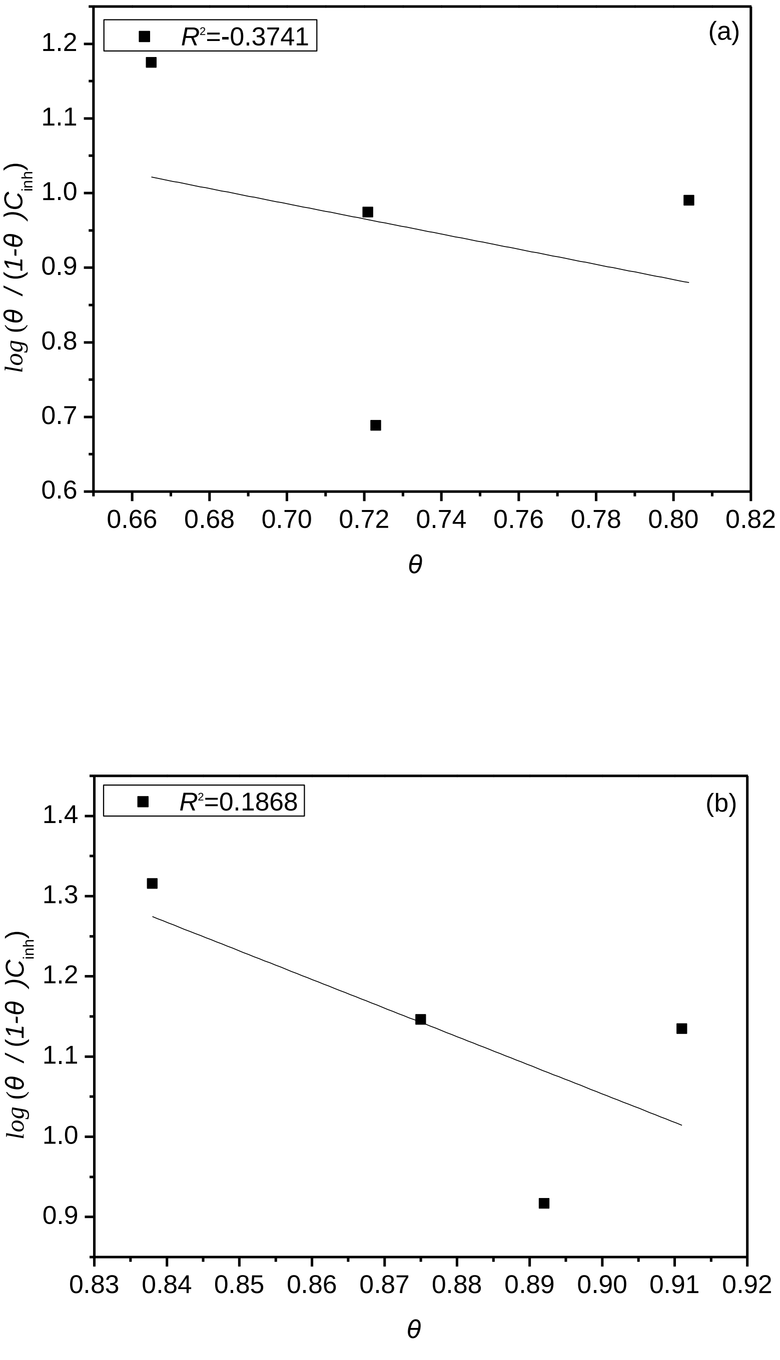

Fig. S9 Frumkin isotherm (log (θ/ (1-θ) C_{inh}) = log K_{ads} + $g\theta$) for carbon steel in 0.5 M HCl solution with different concentrations of BAIL1 (a) and BAIL2 (b) using EIS data

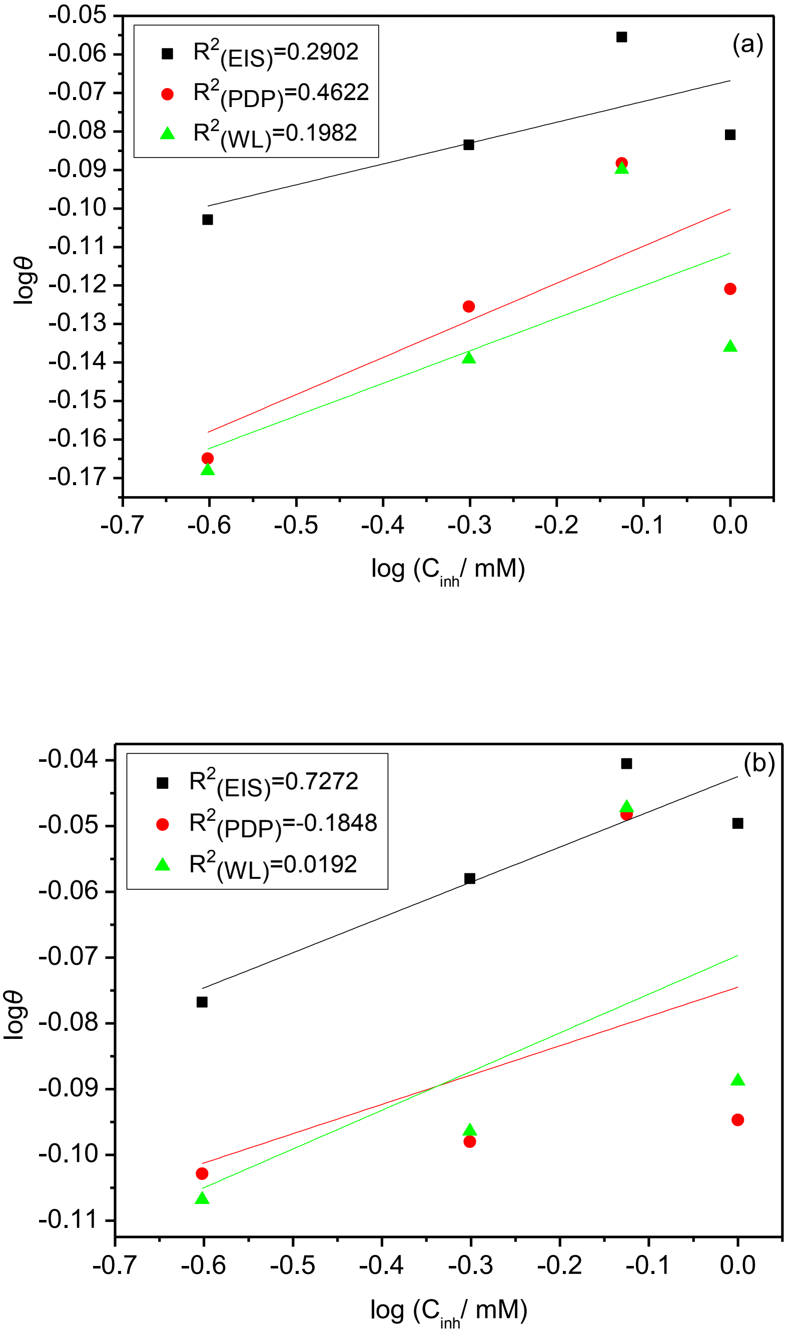

Fig. S10 Freundlich isotherm ($\log \theta = \log K_{ads} + 1/n \log C_{inh}$) for carbon steel in 0.5 M HCl solution with different concentrations of BAIL1 (a) and BAIL2 (b) using EIS data
